# A machine learning approach to predict treatment response in myofascial pain patients receiving masseter trigger point injections

**DOI:** 10.1186/s12903-026-09491-0

**Published:** 2026-08-10

**Authors:** Alshaimaa Ahmed Shabaan, Islam Kassem, Aliaa Ibrahium Mahrous, Inass Aboulmagd, Islam A. Amer, Ahmed Shaaban, Kareem Kamal Fathy, Reham Ragab, Mohamed Abd-El-Ghafour, Sally Ibrahim, Shaimaa Mohsen Refahee

**Affiliations:** 1 Oral & Maxillofacial Surgery Department, Faculty of Dentistry, Fayoum University, Fayoum, Egypt; 2 Oral & Maxillofacial Surgery, Faculty of Dentistry, MUST University, Giza, Egypt; 3 Consultant Oral and Maxillofacial Surgery, Department of Maxillofacial surgery, Alamin Hospital, Ministry of Health & Population, Alamin, Egypt; 4 Consultant Oral and Maxillofacial Surgery, Main University Hospital, Alexandria University, Alexandria, Egypt; 5 Fixed prosthodontic Department, Faculty of Dentistry, Fayoum University, Fayoum, Egypt; 6 Fixed prosthodontic Department, Faculty of Dentistry, MSA University, Giza, Egypt; 7 Oral & Maxillofacial Radiology Department, Faculty of Dentistry, Fayoum University, Fayoum, Egypt; 8 Department of maxillofacial head and neck surgery, faculty of medicine, Sohage University, Sohage, Egypt; 9 Prosthodontic Department, Faculty of Dentistry, Future University, Cairo, Egypt; 10 Nile University, Cairo, Egypt; 11 Research Associate Nile of Hope hospital, Alexandria, Egypt; 12 Department of Orthodontics, Faculty of Dentistry, Cairo University, Cairo, Egypt; 13 Oral & Maxillofacial Pathology Department, Faculty of Dentistry, Fayoum University, Fayoum, Egypt; 14 Oral and Maxillofacial Surgery Department, Faculty of Dentistry, Fayoum University, Fayoum, Egypt

**Keywords:** Myofascial pain syndrome, Trigger point injection, Machine learning, Random forest, XGBoost, Precision medicine, Orofacial pain

## Abstract

**Background:**

Trigger point injection (TPI) therapy is widely used for masseter myofascial pain syndrome (MPS), yet outcomes vary substantially. Individualized prediction tools are lacking, often leading to trial-and-error treatment selection.

**Objective:**

To develop, externally validate, and generalize ensemble machine learning models for predicting composite treatment success following masseter TPI, and to deploy a web-based clinical decision support system (CDSS).

**Methods:**

This multicenter study included 1,181 patients with DC/TMD‑diagnosed masseter MPS treated with one of six injectable modalities. Baseline variables included pain (VAS), maximum mouth opening (MMO), and oral health‑related quality of life (OHIP-14). Composite treatment success was defined as simultaneous clinically meaningful improvement at 3 months: VAS reduction ≥ 2 points, MMO increase ≥ 5 mm, and OHIP-14 reduction ≥ 5 points. Random Forest (RF) and XGBoost models were trained on an internal cohort and externally validated on a geographically independent cohort. Performance was assessed using ROC‑AUC, precision‑recall AUC (PR‑AUC), calibration, decision curve analysis (DCA), and SHAP interpretability.

**Results:**

Overall composite success rate was 43.1%. Internal validation ROC‑AUC was 0.914 (RF) and 0.888 (XGBoost); external validation ROC‑AUC was 0.771 and 0.787, respectively. External PR‑AUC values were 0.759 (RF) and 0.761 (XGBoost). Both models showed good calibration and positive net benefit on DCA across clinically relevant thresholds. SHAP analysis identified baseline MMO, OHIP-14, age, pain intensity, and injectable modality as the most influential predictors, with consistent rankings across models and cohorts. The validated XGBoost model was deployed as a web‑based CDSS.

**Conclusions:**

Machine learning models demonstrated good ability to predict multidimensional treatment success following masseter TPI. Baseline MMO, OHIP-14, age, pain intensity, and injectable modality were the strongest outcome determinants. External validation, SHAP interpretability, and DCA support model robustness and potential clinical utility for personalized treatment planning in MPS.

**Clinical relevance:**

This tool may support treatment selection, reduce ineffective interventions, and improve patient outcomes in myofascial pain management.

**Supplementary Information:**

The online version contains supplementary material available at 10.1186/s12903-026-09491-0.

## Introduction

Myofascial pain syndrome (MPS) is a chronic musculoskeletal condition characterized by localized pain in muscles and associated tissues, primarily due to myofascial trigger points (MTrPs) [1, 2]. MTrPs are hyper-irritable spots in taut bands of skeletal muscle, which often refer to pain to different body areas, leading to significant discomfort. The masseter muscle, one of the main muscles responsible for mastication, is frequently affected by MPS, resulting in pain and functional limitations [3, 4].

Patients with MPS in the masseter muscle often present symptoms including persistent pain, restricted jaw movement, tenderness on palpation, and referred pain patterns that may extend to the temples, ears, and even neck and shoulders [4, 5]. The impact of MPS, especially in the masseter muscle, on quality of life (QoL) is significant. Studies have shown that individuals suffering from MPS experience decreased functioning in daily activities, difficulties with eating and swallowing, and challenges in social interactions due to pain and discomfort in the jaw [6, 7].

Trigger point injection (TPI) therapy is a widely used intervention for managing myofascial pain syndromes, particularly concerning muscles where myofascial trigger points are present. TPI involves the injection of a therapeutic substance directly into these trigger points to alleviate pain and restore function. The efficacy of TPI for MPS has been supported by several randomized controlled trials and systematic reviews. For instance, research shows that TPI notably improves pain levels and function in patients with myofascial pain, regardless of the injectate used [3, 5, 6, 8].

Despite its widespread use, the clinical efficacy of TPI for masseter myofascial pain is characterized by marked heterogeneity in patient outcomes. This variability complicates efforts to establish consistent success rates across different patient populations and clinical settings. The dependence on clinician experience and generalized protocols in administering TPI further amplifies this inconsistency, as practitioners may utilize varying techniques, dosages, and injection sites based on subjective judgments rather than standardized methods [3, 5, 6]. Currently, there is a notable absence of validated tools capable of predicting individual patient responses to TPI. This gap results in clinicians often resorting to trial-and-error methods, which can lead to suboptimal treatment plans and discouraging patient outcomes. An essential requirement for improving TPI practices is the establishment of personalized prediction tools that consider distinct patient parameters while accommodating individual experiences that influence treatment effectiveness [8–10].

Machine learning (ML) has emerged as a transformative technology in healthcare, enabling clinicians to analyze complex data and predict patient outcomes with increasing accuracy. ML has become increasingly established in outpatient pain management across several clinical domains, providing a robust precedent for developing predictive tools for myofascial pain. The neural network and ensemble models have been successfully used to predict postoperative opioid refill needs following ambulatory surgery, achieving AUC values of 0.71–0.75 and identifying actionable predictors such as recovery room pain scores and perioperative opioid exposure [11, 12]. In musculoskeletal outpatient settings, ML models including XGBoost and random forest have demonstrated strong discrimination for predicting rotator cuff tears and guiding referral pathways [13]. Similarly, ML has been applied to outpatient discharge planning after orthopedic procedures such as revision knee arthroplasty for predicting same-day discharge suitability [14, 15].

Through these studies, interpretability analyses using SHAP values have become standard, enhancing clinical transparency. However, several challenges remain, including heterogeneity of outcome definitions, the need for external validation across multiple centers, and ethical considerations regarding privacy and algorithm fairness. Despite these limitations, the established track record of ML in predicting outpatient pain-related outcomes provides a strong methodological foundation and justification for extending this approach to predict treatment response following TPI in masseter myofascial pain [16].

Despite the growing application of machine learning in pain management, predictive models specifically designed for treatment response following trigger point injections for masseter myofascial pain remain scarce. Existing studies have primarily focused on evaluating treatment efficacy rather than developing individualized prediction tools capable of integrating demographic, clinical, functional, and quality-of-life variables. Because treatment success in myofascial pain is inherently multidimensional, machine learning provides a suitable framework for modeling complex interactions among these factors and estimating personalized probabilities of clinically meaningful improvement [17, 18]. Therefore, the aim of the present study was to develop, externally validate, and geographically generalize ensemble machine learning models for predicting composite treatment success following masseter trigger point injections and to deploy a prototype web-based clinical decision support system for individualized treatment probability estimation.

## Material & method

### Study design and data sources

This study pooled individual patient data from six randomized controlled trials evaluating trigger point injection therapies for myofascial temporomandibular pain, conducted across three Oral and Maxillofacial Surgery centers between March and November 2025: Fayoum University Hospital, New Alamein City Hospital, Sohag University Hospital, and a network of private practices. Four trials employed simple randomization and two employed block randomizations. Treatment allocation followed the randomization scheme of the respective source trial and was not determined by clinician discretion at the time of treatment administration.

Ethical approval was granted by the Fayoum University Ethics Committee (FU-SCSRE; Code: EC24177) & Medical Research Committee (MREC) of Faculty of Medicine, Sohag University (Soh-Med-25-10-PD). Prior to enrolment, all participants provided written informed consent for the scientific use of their clinical data and images. The study was performed in accordance with the ethical standards of the Declaration of Helsinki [19].

### Participants

Participants were recruited from the Oral and Maxillofacial Surgery outpatient clinics at three sites (Alamein/Sohag, Fayoum, and private practice). Eligible individuals were adults with orofacial pain persisting for at least 6 months and diagnosed with unilateral or bilateral trigger points in the masseter muscle, with no evidence of other muscular diseases or neuropathies. Participant selection followed predefined inclusion and exclusion criteria. Participants were eligible for inclusion if they met all of the following criteria: (1) Definite diagnosis of myofascial pain with a referral, based on the DC/TMD criteria [20]; (2) the presence of one or more trigger points in the unilateral or bilateral masseter muscle; and (3) no history of any invasive procedures in the related masseter muscle. Participants were excluded if they met any of the following criteria: (1) Factors that can cause pain in the orofacial region other than MTPs (decayed tooth, TMJ internal disorder); (2) Any systemic disease that possibly affects the masticatory system such as rheumatoid arthritis and epilepsy; (3) pregnancy and lactation; and (4) History of surgical intervention in the masseter muscle (e.g., biopsy, tumor resection, or open TMJ surgery), or receipt of any invasive procedure requiring skin/mucosal incision or instrumentation beyond a percutaneous needle injection.

A total of 1,181 eligible patients across the six pooled randomized controlled trials received TPI with one of six injectable modalities, according to the randomization scheme of their respective source trial: Botulinum Toxin Type A (Botox, *n* = 299), Dry Needling (*n* = 200), Local Anesthetic (*n* = 250), Magnesium Sulfate (*n* = 201), Platelet-Rich Fibrin (PRF, *n* = 151), or Physiological Saline (*n* = 80).

### Data collection and variable definition

Baseline data collection encompassed demographic characteristics (age, sex), treatment location, pain intensity measured using a 10-point Visual Analogue Scale (VAS) [21], maximum mouth opening (MMO) measured in millimeters as inter-incisal distance [22], and oral health-related quality of life assessed via the Oral Health Impact Profile-14 (OHIP-14) questionnaire [23]. Treatment-related variables included injected material type. Follow-up assessments at 1-month and 3-month post-intervention recorded VAS, MMO, and OHIP-14 scores, along with documentation of requirement for repeat injection. OHIP-14 scores at one month were missing for a single patient (*n* = 1) owing to a data-entry omission identified during preprocessing; this isolated missing value precluded reliable reporting of the OHIP-14 M1 timepoint and was therefore not used. OHIP-14 at three months and OHIP-14 change-from-baseline, both of which underlie the composite treatment-success definition, were complete for all 1,181 patients and are reported in Table 2 in place of the M1 value.

### Outcome definition

The primary outcome, composite treatment success, required simultaneous achievement of all three of the following criteria at the three-month follow-up: (1) a reduction in pain intensity of ≥ 2 points on the VAS, representing the established minimal clinically important difference (MCID) for pain [24]; (2) an increase in MMO of ≥ 5 mm, consistent with the DC/TMD functional threshold for clinically meaningful improvement [20]; and (3) a reduction in OHIP-14 score of ≥ 5 points, reflecting the validated MCID for oral health-related quality of life [25]. This AND-logic composite endpoint was selected to ensure that treatment success reflected simultaneous improvement in pain, function, and quality of life rather than isolated change in a single domain.

### Statistical analysis

Baseline demographic and clinical characteristics were summarized as median with interquartile range (IQR) for continuous variables, and as frequency with percentage for categorical variables.

Prior to between-group comparisons, the normality of all continuous variables was formally assessed using the Shapiro–Wilk test, supplemented by examination of skewness values, excess kurtosis, and quantile–quantile plots. All continuous variables demonstrated statistically significant departure from normality (Shapiro–Wilk *p* < 0.001 for all), with skewness values ranging from − 6.41 to + 6.76, confirming the appropriateness of non-parametric inference throughout.

Between-group comparisons of continuous variables were performed using the Kruskal–Wallis H test. Pairwise post-hoc comparisons were conducted using Dunn’s test with Bonferroni correction for multiple testing; a total of 17 significant pairs were identified in baseline comparisons (Table 1) and 77 in outcome comparisons (Table 2). For categorical variables, Pearson’s chi-square test was applied. All contingency cells satisfied the minimum expected frequency criterion (expected count ≥ 5 in all cells), confirming the validity of the chi-square large-sample approximation in this cohort (*N* = 1,181); Monte Carlo permutation testing was therefore not required.

**Table 1:** Baseline characteristics by injectable agent (*N* = 1,181)

Characteristic	Overall *N* = 1,181^*1*^	Botox *N* = 299^*1*^	Dry Needling *N* = 200^*1*^	Local Anaesthesia *N* = 250^*1*^	Magnesium Sulfate *N* = 201^*1*^	PRF *N *= 151^*1*^	Saline *N* = 80^*1*^	*p*-value^*2*^	Effect Size^*3*^
Age, years	34.0 [25.0–41.0]	33.0 [25.0–36.0]	34.0 [25.0–39.5]	32.0 [25.0–39.0]	35.0 [27.0–47.0]	35.0 [28.0–47.0]	33.0 [25.0–42.0]	<0.001	0.019 [ε²]
Sex								<0.001	0.149 [V]
Female	1,092 (92%)	288 (96%)	189 (95%)	223 (89%)	173 (86%)	141 (93%)	78 (98%)		
Male	89 (7.5%)	11 (3.7%)	11 (5.5%)	27 (11%)	28 (14%)	10 (6.6%)	2 (2.5%)		
Centre								<0.001	0.361 [V]
Fayoum University Hospital	542 (46%)	108 (36%)	108 (54%)	124 (50%)	127 (63%)	14 (9.3%)	61 (76%)		
Other Centres	639 (54%)	191 (64%)	92 (46%)	126 (50%)	74 (37%)	137 (91%)	19 (24%)		
Previous injection	660 (56%)	282 (94%)	18 (9.0%)	22 (8.8%)	193 (96%)	139 (92%)	6 (7.5%)	<0.001	0.858 [V]
Baseline Pain VAS (0–10)	7.0 [6.0–8.0]	7.0 [6.0–8.0]	7.0 [7.0–8.0]	7.0 [7.0–8.0]	8.0 [6.0–8.0]	7.0 [6.0–8.0]	7.0 [6.0–8.0]	0.409	<0.001 [ε²]
Baseline MMO, mm	30.7 [30.1–32.0]	30.9 [30.4–32.0]	30.7 [30.0–31.5]	30.9 [30.3–32.0]	30.5 [29.7–31.0]	30.9 [29.7–32.8]	31.1 [30.5–32.5]	<0.001	0.028 [ε²]
Baseline OHIP-14 (0–56)	39.0 [36.0–42.0]	37.0 [35.0–39.0]	40.0 [37.0–44.0]	37.0 [35.0–41.0]	42.0 [39.0–42.0]	40.0 [38.0–40.0]	37.0 [36.0–42.0]	<0.001	0.136 [ε²]

^1^Median [Q1–Q3]; *n* (%)

^2^Kruskal-Wallis test for continuous variables; Chi-square test for categorical variables. Post-hoc pairwise comparisons performed using Dunn test with Bonferroni correction

^3^ε²: epsilon-squared (small ≥0.01, medium ≥0.06, large ≥0.14). V: Cramér's V (small ≥0.10, medium ≥0.30, large ≥0.50). Values: Median [IQR] or *n* (%)

**Table 2:** Treatment outcomes at 1-month and 3-month follow-up by injectable agent (*N* = 1,181)

Characteristic	Overall *N* = 1,181^*1*^	Botox *N* = 299^*1*^	Dry Needling *N* = 200^*1*^	Local Anesthesia *N* = 250^*1*^	Magnesium Sulfate *N* = 201^*1*^	PRF *N* = 151^*1*^	Saline *N* = 80^*1*^	*p*-value^2^	Effect Size^3^
Required 2nd injection	63 (5.3%)	1 (0.3%)	6 (3.0%)	38 (15%)	0 (0%)	0 (0%)	18 (23%)	**< 0.001**	0.334 [V]
Treatment outcome								**< 0.001**	0.47 [V]
Treatment success	509 (43%)	70 (23%)	73 (37%)	52 (21%)	158 (79%)	103 (68%)	53 (66%)		
Treatment failure	672 (57%)	229 (77%)	127 (64%)	198 (79%)	43 (21%)	48 (32%)	27 (34%)		
Pain VAS at 1-month	1.0 [0.0–1.0]	0.0 [0.0–1.0]	1.0 [1.0–1.0]	1.0 [0.0–1.0]	0.0 [0.0–0.0]	1.0 [0.0–1.0]	1.0 [1.0–1.0]	**< 0.001**	0.32 [ε²]
Pain VAS at 3-month	0.0 [0.0–1.0]	0.0 [0.0–0.0]	1.0 [1.0–1.0]	1.0 [0.0–2.0]	0.0 [0.0–0.0]	0.0 [0.0–0.0]	1.0 [1.0–1.0]	**< 0.001**	0.535 [ε²]
Pain reduction (M0–M3)	7.0 [6.0–8.0]	7.0 [6.0–8.0]	6.0 [6.0–7.0]	6.0 [5.0–7.0]	7.0 [6.0–8.0]	7.0 [6.0–8.0]	6.0 [5.0–7.0]	**< 0.001**	0.181 [ε²]
MMO at 1-month, mm	36.8 [34.1–38.2]	34.5 [33.7–36.6]	36.9 [34.0–38.3]	34.2 [33.6–35.6]	38.1 [37.1–39.1]	38.1 [36.9–38.6]	37.9 [37.6–39.4]	**< 0.001**	0.407 [ε²]
MMO at 3-month, mm	35.6 [33.4–37.1]	33.9 [32.8–35.6]	34.6 [33.3–36.3]	33.7 [32.7–35.4]	37.1 [35.9–37.8]	36.9 [36.1–38.1]	36.9 [36.3–38.2]	**< 0.001**	0.364 [ε²]
MMO increase (M3–M0), mm	4.5 [2.4–6.3]	2.9 [1.7–4.9]	4.1 [2.2–5.5]	2.8 [1.6–4.5]	6.4 [5.2–7.7]	5.9 [4.6–7.6]	5.7 [4.5–7.1]	**< 0.001**	0.29 [ε²]
OHIP-14 at 3-month	2.0 [0.0–2.0]	2.0 [1.0–3.0]	1.5 [0.0–2.0]	2.0 [0.0–3.0]	1.0 [0.0–2.0]	2.0 [0.0–2.0]	2.5 [1.0–3.0]	**< 0.001**	0.018 [ε²]
OHIP-14 reduction (M0–M3)	37.0 [35.0–40.0]	36.0 [34.0–38.0]	40.0 [36.0–42.0]	35.0 [32.0–39.0]	39.0 [37.0–41.0]	38.0 [36.0–40.0]	35.0 [33.5–39.0]	**< 0.001**	0.153 [ε²]

OHIP-14 at 1 month (M1) was missing for one patient (*n* = 1) due to a data-entry omission identified during preprocessing; OHIP-14 at three months and OHIP-14 change-from-baseline (used in the composite outcome definition) were complete for all 1,181 patients and are reported in place of the M1 value

^1^*n* (%); Median [Q1–Q3]

^2^Kruskal-Wallis test for continuous; Chi-square test for categorical. Post-hoc pairwise comparisons performed using Dunn test with Bonferroni correction.

^3^ε²: epsilon-squared (small ≥ 0.01, medium ≥ 0.06, large ≥ 0.14). V: Cramér’s V (small ≥ 0.10, medium ≥ 0.30, large ≥ 0.50). Overall treatment success: 43.1% (95% Wilson CI: 40.3–45.9%). Success = pain VAS reduction ≥ 2 pts AND MMO increase ≥ 5 mm AND OHIP-14 reduction ≥ 5 pts. Values: Median [IQR] or *n* (%).

Effect sizes were reported for all comparisons to quantify the magnitude of between-group differences beyond statistical significance. For Kruskal–Wallis tests, epsilon-squared (ε²) was used as the effect size estimator, interpreted according to Cohen (1988) benchmarks: small (ε² < 0.01), medium (0.01–0.06), and large (> 0.06) — noting that for Kruskal–Wallis ε², the conventional large threshold is ≥ 0.14 in applied clinical research. For chi-square tests, Cramér’s V was computed and interpreted as small (< 0.10), medium (0.10–0.30), or large (> 0.50) per Cohen (1988).

Composite treatment success rates and their 95% confidence intervals were estimated using the Wilson score method, which provides superior coverage accuracy relative to the Wald method, particularly for proportions departing from 0.50. The overall success rate was 43.1% (95% Wilson CI: 40.3–45.9%). Inter-group differences in success rates were tested using Pearson’s chi-square test with Cramér’s V as the effect size measure.

Statistical significance was defined as a two-sided *p* < 0.05 throughout. All statistical analyses were performed in R version 4.6.0 (R Foundation for Statistical Computing, Vienna, Austria), using the gtsummary and rstatix packages.

### Machine learning model development

Two ensemble machine learning classifiers; Random Forest (RF) and Extreme Gradient Boosting (XGBoost, version 3.0.5) were developed and compared for prediction of composite treatment success. Model development and reporting followed the TRIPOD + AI framework [26].

To account for baseline heterogeneity arising from the pooled multi-trial design, all available demographic, clinical, and treatment-related variables were included as candidate predictors during model development.

#### Dataset partitioning

A geographically stratified validation design was adopted in preference to random cross-validation, to assess model generalizability to independent clinical sites. Patients from Fayoum University Hospital (*n* = 493) constituted the internal dataset and were split into training (80%, *n* = 438) and internal test (20%, *n* = 55) sets using stratified random sampling to preserve outcome class distribution. Patients from New Alamein City Hospital, Sohag University Hospital, and private practices (*n* = 639) formed the geographically independent external validation cohort.

#### Preprocessing

Preprocessing. A preprocessing pipeline was applied exclusively to the training set and subsequently applied without refitting to the internal test and external validation sets. All preprocessing operations, including imputation, standardization, and categorical encoding, were fitted exclusively on the training dataset and then applied unchanged to the validation cohorts, thereby preventing information leakage from validation data into model development. Numeric features underwent median imputation followed by standardization (zero mean, unit variance), while multi-level categorical features were processed using one-hot encoding. All preprocessing was implemented using scikit-learn version 1.7.2.

#### Hyperparameter optimization

Both models underwent hyperparameter tuning via 5-fold stratified cross-validation grid search on the training set, with ROC-AUC as the optimization criterion. For RF, tuned parameters included number of estimators, maximum tree depth, minimum samples per split, and minimum samples per leaf. For XGBoost, optimized parameters comprised learning rate, maximum depth, number of estimators, subsample ratio, column subsampling ratio, and scale_pos_weight (adjusted to address class imbalance in the training set).

Classification Threshold. Optimal probability classification thresholds were determined separately for each model using Youden’s Index (J = sensitivity + specificity − 1), estimated on the internal test set. All reported sensitivity, specificity, F1, and accuracy values correspond to these Youden-optimal thresholds.

#### Model evaluation

Discriminative performance was quantified using ROC-AUC with 95% confidence intervals estimated via the DeLong method. Between-model AUC differences were formally tested using the DeLong test for correlated ROC curves. Precision-recall AUC (PR-AUC) was reported as a supplementary discrimination metric given the 43.1% success prevalence in this cohort. Calibration was assessed using the Brier score (lower scores indicate better calibration). Classification performance at the optimal threshold was summarized by sensitivity, specificity, F1-score, and accuracy. Clinical utility was evaluated using Decision Curve Analysis (DCA), which quantifies net benefit across the full range of threshold probabilities and benchmarks model-guided decisions against the “treat all” and “treat none” reference strategies.

#### Interpretability analysis

Model predictions were interpreted using SHapley Additive exPlanations (SHAP), implemented via the TreeExplainer algorithm. Population-level feature importance was visualized using beeswarm plots displaying the distribution of SHAP values across all patients in each validation set (Figs. 2 and 3). Because TreeSHAP explanations are model-specific and depend on each model’s prediction function and output scale, absolute SHAP magnitudes are not directly comparable between Random Forest and XGBoost. Therefore, cross-model interpretation focused on the relative ranking of predictors, the direction of feature contributions, and the Spearman correlation between feature rankings rather than on numerical SHAP magnitudes. Individual-level prediction reasoning was illustrated for the two most extreme predictions in the external cohort using waterfall plots (Fig. 4). Concordance between RF and XGBoost feature importance rankings was quantified using Spearman’s rank correlation coefficient.

To facilitate prospective clinical application of the developed models, a web-based clinical decision support system (CDSS) was deployed as a publicly accessible interface. The tool accepts individual patient baseline characteristics as inputs and returns the predicted probability of composite treatment success alongside contributing SHAP feature values, enabling point-of-care interpretability.

The CDSS is available at: https://huggingface.co/spaces/drkareemkamal/tmj-predictor [accessed May 2026].

## Results

### Baseline characteristics

A total of 1,181 patients were enrolled across six treatment groups: Botox (*n* = 299), Dry Needling (*n* = 200), Local Anesthesia (*n* = 250), Magnesium Sulfate (*n* = 201), PRF (*n* = 151), and Saline (*n* = 80) (Table 1). The overall median age was 34 years [IQR: 25–41], with no clinically meaningful difference across groups despite reaching statistical significance (*p* < 0.001, ε² = 0.019) — a pattern better attributed to the large sample size than any genuine clinical disparity. Post-hoc comparisons identified three significant age pairs: Botox vs. Magnesium Sulfate, Botox vs. PRF, and Local Anesthesia vs. PRF (all p.adj < 0.05), though the differences in absolute terms were modest. The cohort was predominantly female (92%), with sex distribution varying from 98% in the Saline group to 86% in the Magnesium Sulfate group (*p* < 0.001, Cramér’s V = 0.149).

Centre distribution was expectedly uneven (*p* < 0.001, V = 0.361), reflecting the protocol-to-site associations inherent in a multi-center design; Fayoum University Hospital contributed 76% of Saline patients yet only 9% of PRF patients, against an overall site contribution of 46%. Prior injection history showed the starkest group-level imbalance of any baseline variable (*p* < 0.001, V = 0.858): Botox (94%), Magnesium Sulfate (96%), and PRF (92%) patients had almost universally received a previous injection, compared with fewer than 10% in the Local Anesthesia and Saline groups. This near-perfect separation most plausibly reflects clinical treatment sequencing rather than randomization failure, and should be factored into any cross-group outcome comparisons.

Baseline pain VAS scores were broadly similar across groups, with a shared overall median of 7 [IQR: 6–8] and no statistically significant between-group difference (*p* = 0.409, ε² < 0.001) — a reassuring finding that suggests reasonable pain-level comparability at enrolment. Baseline MMO showed a small but statistically significant difference (*p* < 0.001, ε² = 0.028), driven primarily by post-hoc contrasts involving Magnesium Sulfate and Saline; the overall median was 30.7 mm [IQR: 30.1–32.0], a range clustering tightly around the lower boundary of normal mouth opening. Baseline OHIP-14 carried the most clinically meaningful between-group variation (*p* < 0.001, ε² = 0.136, medium effect), with post-hoc testing identifying eight significant pairs — most notably Dry Needling vs. Local anesthesia (p.adj < 0.001) and Local Anesthesia vs. Magnesium Sulfate (p.adj < 0.001). The Magnesium Sulfate group entered the study with the highest median quality-of-life impairment (42 [IQR: 39–42]), 5 points above the Botox group (37 [IQR: 35–39]), a difference that warrants attention when interpreting downstream OHIP-14 outcomes.

### Treatment outcomes

Overall, 509 patients met the composite success criterion, yielding a success rate of 43.1% (95% Wilson CI: 40.3–45.9%), though this figure masks striking heterogeneity across groups (*p* < 0.001, V = 0.47, large effect). Magnesium Sulfate and PRF performed most strongly, with success rates of 79% and 68% respectively, while Botox (23%) and Local Anesthesia (21%) fell well below the cohort average. Saline (66%) and Dry Needling (37%) occupied intermediate positions. The need for re-injection reinforced this hierarchy: 23% of Saline patients and 15% of those receiving Local Anesthesia required a second procedure, against zero re-injections in both the Magnesium Sulfate and PRF groups (*p* < 0.001, V = 0.334, moderate-to-large effect) (Table 2).

To better characterize treatment failures, the non-responder group (*n* = 672; 56.9%) was examined descriptively. Failure rates varied substantially according to injectable material. The highest proportions of non-response were observed in the Local Anesthesia group (79.2%, 198/250) and Botox group (76.6%, 229/299), whereas Magnesium Sulfate (21.4%, 43/201) and PRF (31.8%, 48/151) demonstrated considerably lower failure rates. Comparison of baseline clinical characteristics revealed only minimal differences between responders and non-responders. Median baseline pain intensity (VAS) was identical in both groups (7.0), while baseline maximum mouth opening differed by less than 1 mm (31.2 mm versus 30.4 mm). These findings suggest that baseline severity measures alone do not adequately explain treatment failure and support the need for multivariable machine learning approaches capable of integrating multiple clinical predictors to estimate individualized treatment response.

Pain outcomes at one month already separated the groups substantially (*p* < 0.001, ε² = 0.32, large effect), with a median VAS of 0 [0–0] in Magnesium Sulfate versus 1 [1–1] in Dry Needling and Saline. By three months the divergence deepened further (ε² = 0.535, large effect): Botox and Magnesium Sulfate converged on a median of 0 [0–0], while Local Anesthesia patients still registered a median of 1 [0–2], with a notable tail persisting into scores 2–3. Post-hoc comparisons for three-month pain identified nine significant pairs — Botox significantly outperformed Dry Needling, Local Anesthesia, and Saline (all p.adj < 0.001), as did Magnesium Sulfate and PRF versus Dry Needling and Saline. Overall pain reduction across the cohort reached a median of 7 points [IQR: 6–8] (ε² = 0.181), with Local Anesthesia and Saline achieving the lowest median reductions at 6 points [5–7]; post-hoc testing confirmed that both groups reduced pain significantly less than Magnesium Sulfate and PRF (all p.adj < 0.001) (Table 2).

Mouth opening improvements followed a similarly differentiated pattern. At one month, Magnesium Sulfate and PRF recorded the highest median MMO values — 38.1 mm [IQR: 37.1–39.1] and 38.1 mm [36.9–38.6] respectively — compared with 34.2 mm [33.6–35.6] in Local Anesthesia (*p* < 0.001, ε² = 0.407, large effect), with post-hoc contrasts confirming that Local Anesthesia was significantly inferior to all other groups except Dry Needling. By three months, the absolute MMO gains told the clearest story: Magnesium Sulfate achieved a median increase of 6.4 mm [IQR: 5.2–7.7] and PRF 5.9 mm [4.6–7.6], both exceeding the 5 mm MCID threshold, while Botox (2.9 mm [1.7–4.9]) and Local Anesthesia (2.8 mm [1.6–4.5]) fell clearly below it (*p* < 0.001, ε² = 0.29, large effect). This dissociation — strong pain control in Botox alongside limited mouth opening improvement — is a clinically meaningful finding that likely reflects the different mechanisms of action involved (Table 2).

Quality-of-life recovery, measured by OHIP-14 reduction from baseline, showed a medium-to-large between-group effect (*p* < 0.001, ε² = 0.153). Dry Needling produced the largest median reduction (40 points [IQR: 36–42]), followed by Magnesium Sulfate (39 points [37–41]) and PRF (38 points [36–40]); Local Anesthesia and Saline recorded the smallest reductions at 35 points [32–39] and 35 points [33.5–39] respectively, with post-hoc testing confirming significant differences between Dry Needling and Local Anesthesia (p.adj < 0.001) and between Local Anesthesia and Magnesium Sulfate (p.adj < 0.001). Residual OHIP-14 scores at three months approached near-complete resolution across most groups (overall median 2 [IQR: 0–2]), though Saline patients retained slightly higher residual impairment (2.5 [1–3]) and were significantly worse than every other group in pairwise comparisons (all p.adj < 0.05) (Table 2).

### Predictive modelling of treatment success

Two machine learning classifiers — Random Forest (RF) and XGBoost (XGB) were trained to predict composite treatment success from baseline patient characteristics and injection material assignment. Both models achieved excellent discrimination on the training data, with ROC-AUC values of 0.986 and 0.970 for RF and XGB respectively, and correspondingly low Brier scores (0.068 and 0.071), indicating well-calibrated probability estimates during training (Table 3).

**Table 3:** Predictive model performance for composite treatment success across training, internal validation, and external validation sets

Model	Dataset	N	ROC-AUC	95% CI	PR-AUC	Brier Score	Sensitivity	Specificity	F1	Accuracy
Random Forest	Train	438	0.986	[0.978–0.993]	0.984	0.068	0.923	0.967	0.940	0.947
Random Forest	Internal Test	55	0.914	[0.843–0.985]	0.888	0.129	0.917	0.806	0.846	0.855
Random Forest	External	639	0.771	[0.733–0.809]	0.759	0.183	0.554	0.927	0.670	0.770
XGBoost	Train	438	0.970	[0.956–0.983]	0.966	0.071	0.903	0.926	0.905	0.916
XGBoost	Internal Test	55	0.888	[0.806–0.971]	0.873	0.139	0.917	0.710	0.800	0.800
XGBoost	External	639	0.787	[0.751–0.822]	0.761	0.189	0.502	0.932	0.629	0.751

The composite success criterion was defined as simultaneous achievement of pain VAS reduction ≥ 2 points, MMO increase ≥ 5 mm, and OHIP-14 reduction ≥ 5 points. Models were trained on patients from the Fayoum cohort (internal set, *N* = 542; 80% train / 10% validation / 10% test via stratified split) and externally validated on all remaining sites (*N* = 639), constituting a geographically independent validation cohort. The scale_pos_weight parameter for XGBoost was set to 1.246 to account for the class imbalance (43.2% success rate). 95% confidence intervals were computed using the DeLong structural components method

*Abbreviations*: *ROC-AUC* area under the receiver operating characteristic curve, *PR-AUC* area under the precision-recall curve, *Brier* Brier score (lower is better), *F1* harmonic mean of precision and recall, *RF* Random Forest, *XGB* XGBoost

**Fig. 1:**
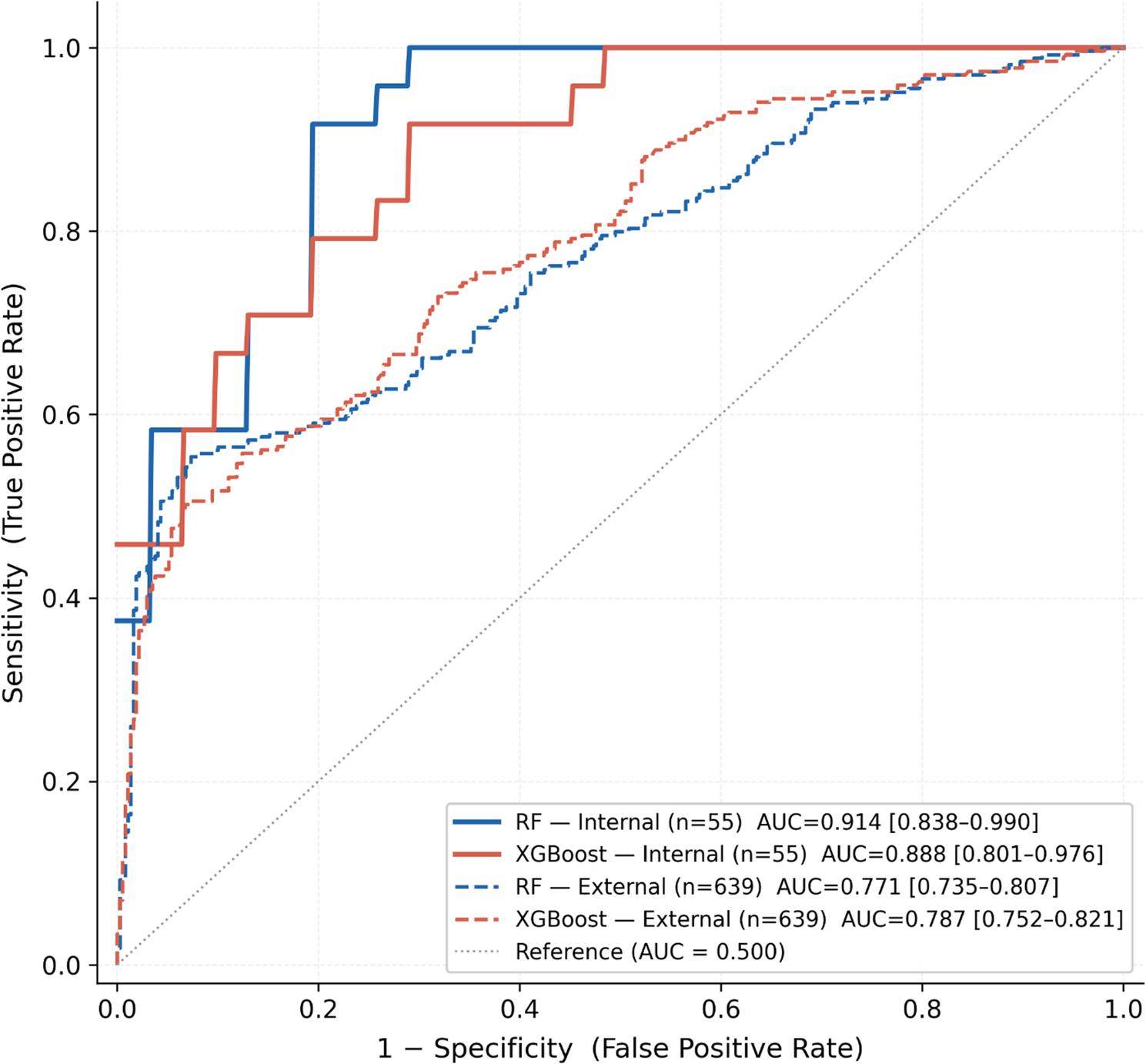
Receiver operating characteristic (ROC) curves for Random Forest and XGBoost classifiers across internal and external validation cohorts. Solid lines represent internal validation (Fayoum University Hospital, n = 55); dashed lines represent external validation (New Alamein City Hospital, Sohag University Hospital, and Private Practices, n = 639). Blue = Random Forest; red/orange = XGBoost

Internal validation on the held-out test set (*n* = 55) confirmed that discriminative performance was largely preserved: RF retained an AUC of 0.914 (95% CI: 0.843–0.985) and XGB 0.888 (95% CI: 0.806–0.971), both remaining in the excellent range. Sensitivity was high in both models at 91.7%, though specificity declined modestly — more so for XGB (71.0%) than RF (80.6%) suggeing a degree of optimism that wider external validation was necessary to address. The between-model AUC difference was not statistically significant by the DeLong test (stinternal: *p* = 0.236) (Table 3; Fig. 1).

External validation across the independent cohort (*n* = 639) revealed the expected attenuation in performance, with RF and XGB achieving ROC-AUC values of 0.771 (95% CI: 0.733–0.809) and 0.787 (95% CI: 0.751–0.822) respectively, both remaining in the acceptable-to-good range. The between-model difference was again not statistically significant by the DeLong test (external: *p* = 0.110), indicating comparable discriminative performance between classifiers across both cohorts. The most notable shift in external validation was in sensitivity, which fell to 55.4% for RF and 50.2% for XGB, while specificity remained strong (92.7% and 93.2% respectively), indicating that both models were conservative in flagging success — they rarely misclassified a failure as a success, but missed a meaningful proportion of true successes. PR-AUC values in external validation (0.759 for RF and 0.761 for XGB) are particularly informative given the 43.2% success prevalence in this cohort, confirming that precision-recall performance remained well above the no-skill baseline of 0.432. Taken together, these findings support the clinical utility of both classifiers as screening tools to identify patients unlikely to achieve composite success, while acknowledging that external generalizability warrants prospective validation in independent TMJ populations (Table 3; Fig. 1).

Visual inspection of the ROC curves demonstrated the expected attenuation in model performance when moving from internal to external validation (Fig. 1). Nevertheless, both Random Forest and XGBoost maintained acceptable-to-good discrimination in the geographically independent cohort, with ROC curves remaining substantially above the no-discrimination reference line (AUC = 0.500) across the full range of specificity values. The separation between the internal and external validation curves reflects the anticipated reduction in performance when models are evaluated in previously unseen clinical settings, providing a more realistic estimate of generalizability. Importantly, no statistically significant difference in discriminative performance was observed between the two algorithms in either the internal validation cohort (DeLong *p* = 0.236) or the external validation cohort (DeLong *p* = 0.110), indicating comparable classification capability across both validation settings.

### SHAP interpretability

#### Internal validation cohort

**Fig. 2:**
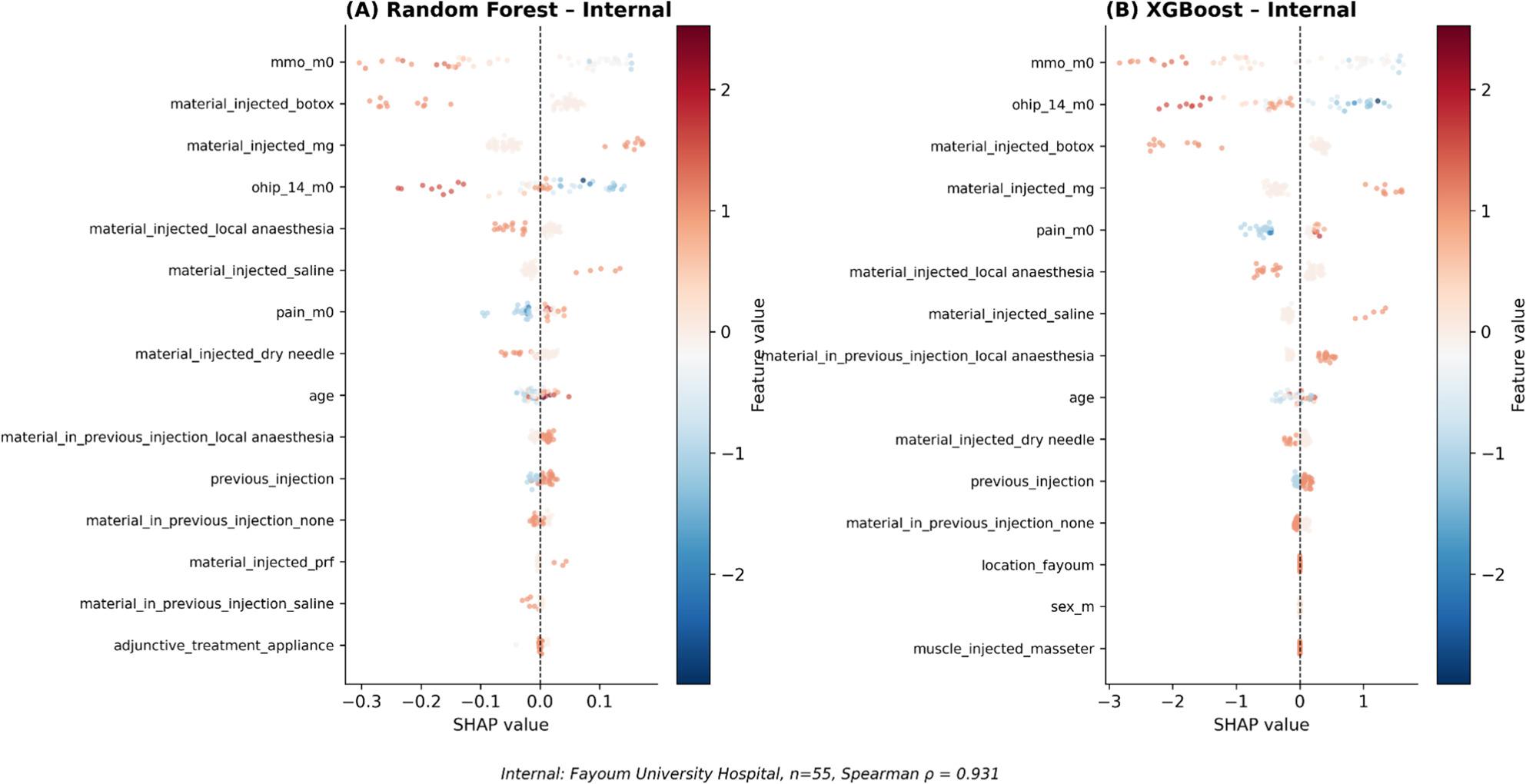
SHAP beeswarm plots for the internal validation cohort (Fayoum University Hospital, *n* = 55)

Because SHAP values are model-specific, the larger numerical SHAP values observed for XGBoost compared with Random Forest reflect differences in model output scale rather than greater predictor importance. Accordingly, interpretation focuses on the consistency of feature rankings and contribution directions rather than absolute SHAP magnitudes. In Fig. 2, SHAP analysis of the internal validation set (Fayoum University Hospital, *n* = 55) identifies baseline MMO as the single most influential predictor in both models, ranking first in both Random Forest (mean |SHAP| = 0.127) and XGBoost (mean |SHAP| = 1.254). Higher baseline mouth opening was associated with a negative SHAP contribution — a counterintuitive direction that likely reflects a ceiling effect: patients who already approach a normal range have less physiological room to register a ≥ 5 mm gain meeting the MCID threshold. Injection material assignment contributed the next largest influence: Botox consistently exerted a negative SHAP contribution regardless of feature value (RF rank 2, mean |SHAP| = 0.085; XGB rank 3, mean |SHAP| = 0.656), while Magnesium Sulfate showed a consistently positive directional effect (RF rank 3, mean |SHAP| = 0.075; XGB rank 4, mean |SHAP| = 0.554), mirroring the outcome hierarchy observed in Table 2. Baseline OHIP-14 score ranked fourth in Random Forest (mean |SHAP| = 0.069) but rose to second in XGBoost (mean |SHAP| = 0.822), with higher baseline impairment generally predicting lower success probability — consistent with the observation that the Magnesium Sulfate’s group entered the study carrying the heaviest quality-of-life burden. Feature importance rankings between the two models were highly concordant in this cohort (Spearman ρ = 0.931, *p* < 0.001), lending confidence that the identified predictors reflect genuine signal rather than model-specific artefact.

#### External validation cohort

**Fig. 3:**
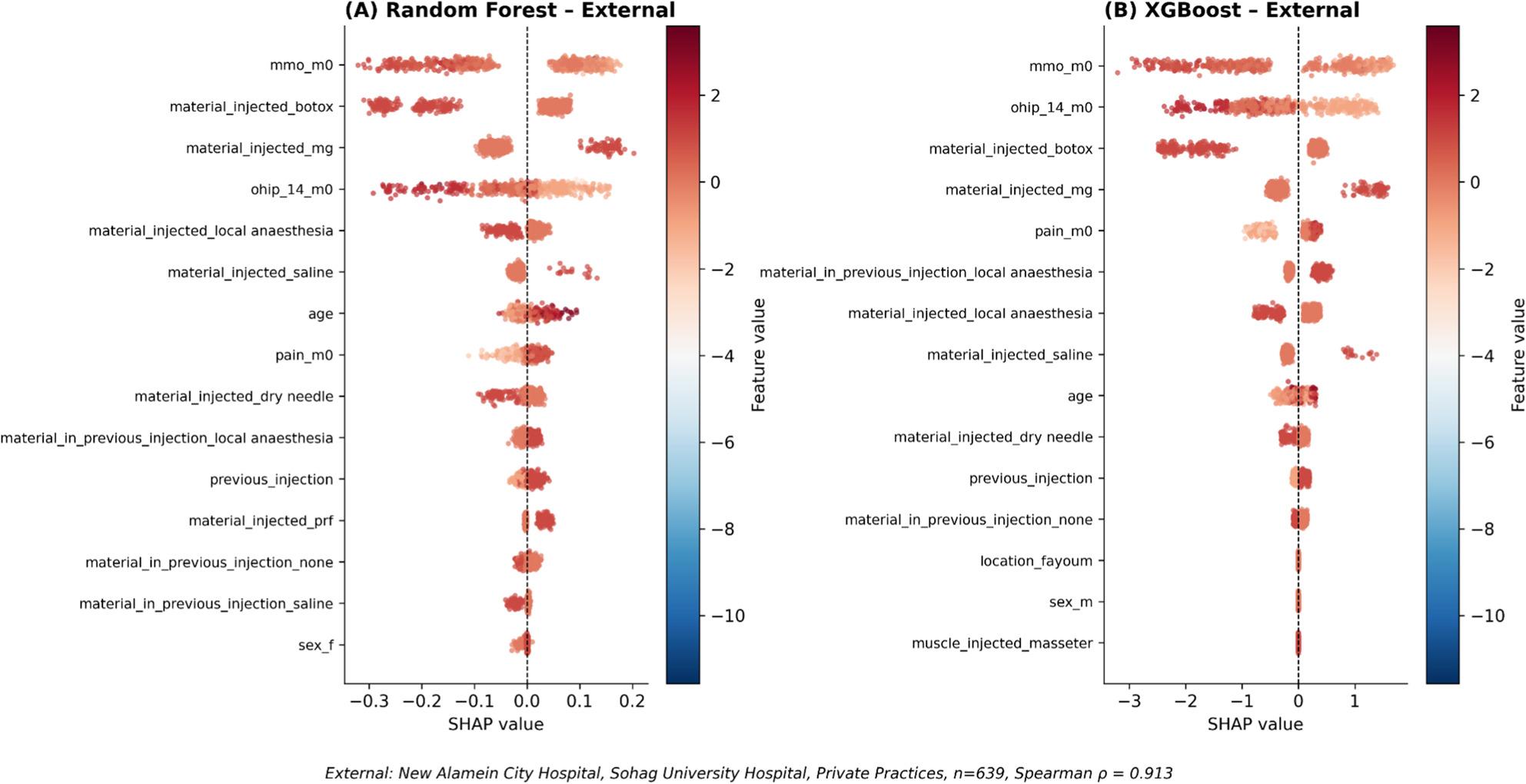
SHAP beeswarm plots for the external validation cohort (New Alamein City Hospital, Sohag University Hospital, and Private Practices, *n* = 639)

In the external cohort (New Alamein City Hospital, Sohag University Hospital, and Private Practices; *n* = 639), the feature importance structure was remarkably stable across sites (Fig. 3). Baseline MMO retained the top rank in both models (RF mean |SHAP| = 0.127; XGB mean |SHAP| = 1.226), with a consistent negative directional effect mirroring that of the internal cohort — a strong indicator that this predictor generalizes across geographically and institutionally distinct patient populations. Botox assignment retained its consistently negative SHAP contribution (RF rank 2, mean |SHAP| = 0.101; XGB rank 3, mean |SHAP| = 0.811), and Magnesium Sulfate its positive one (RF rank 3, mean |SHAP| = 0.071; XGB rank 4, mean |SHAP| = 0.485), reinforcing the clinical interpretation that injection material is a modifiable and predictable driver of treatment success. Baseline OHIP-14 ranked fourth in Random Forest (mean |SHAP| = 0.065) and second in XGBoost (mean |SHAP| = 0.838) in the external cohort, with a wider spread of SHAP values than observed internally, likely reflecting the greater baseline OHIP-14 heterogeneity present across multiple sites. Baseline pain VAS gained relative prominence in the external set, ranking fifth in XGBoost (mean |SHAP| = 0.310), suggesting that in a more heterogeneous population pre-treatment pain severity carries additional discriminative value beyond what the more homogeneous Fayoum cohort alone could reveal. Cross-model rank correlation confirmed the stability of these patterns (Spearman ρ = 0.913, *p* < 0.001), and cross-cohort rank correlations further indicated strong generalizability of the predictor structure: RF ρ = 0.997 and XGB ρ = 0.993 (both *p* < 0.001) (Fig. 3).

#### Individual-level prediction reasoning

**Fig. 4:**
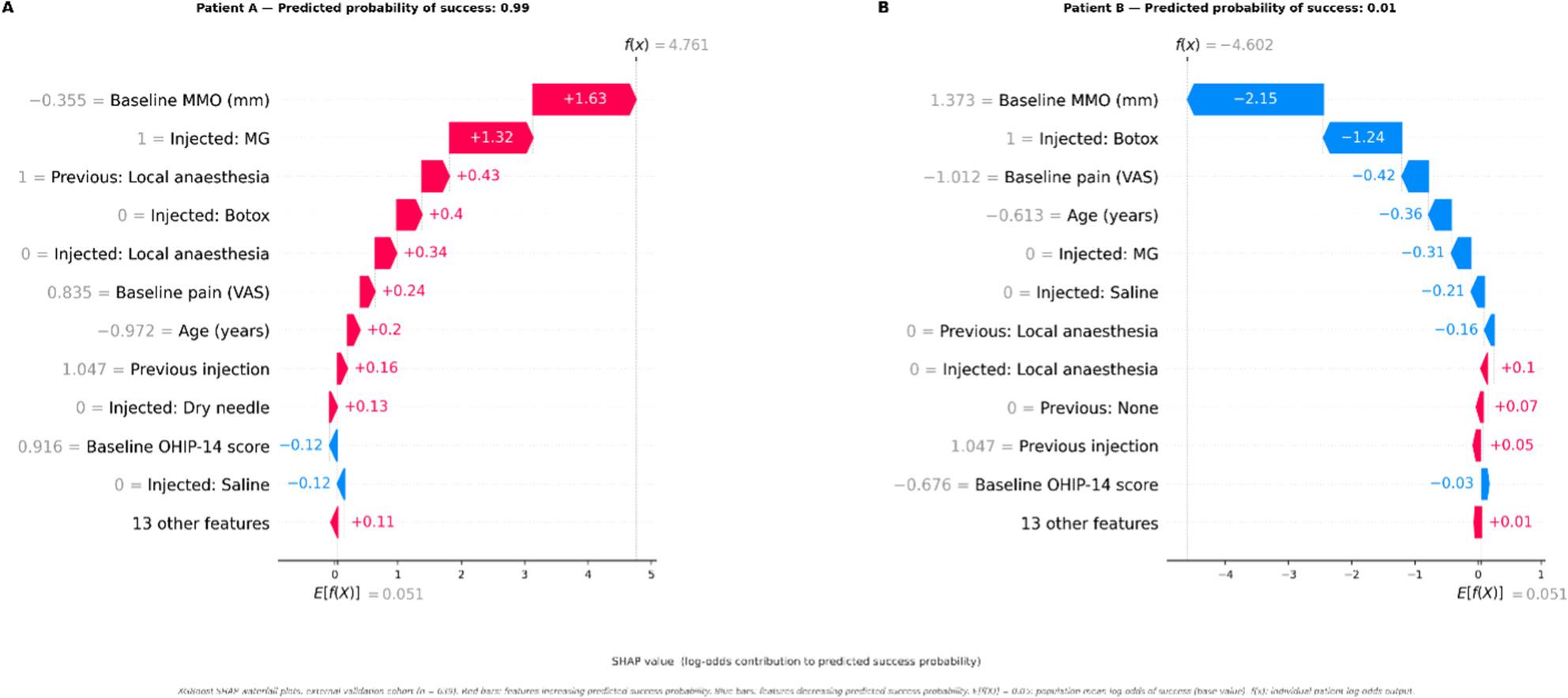
XGBoost SHAP waterfall plots illustrate individual-level prediction reasoning for two representative patients from the external validation cohort (*n* = 639)

To ground the population-level SHAP findings in individual clinical terms, waterfall plots were generated for the two most extreme predictions produced by XGBoost in the external cohort (Fig. 4). Patient A (predicted probability of success = 0.99; f(x) = 4.761) received Magnesium Sulfate injection — the second largest positive contributor (+ 1.32 log-odds units) — and presented with a below-average baseline MMO (scaled value = − 0.355), which the model interpreted as the single strongest positive signal (+ 1.63), consistent with greater physiological capacity to register a ≥ 5 mm mouth-opening gain against the MCID threshold. A history of prior local anesthesia injection further promoted the prediction (+ 0.43), followed by the absence of Botox assignment (+ 0.40) and the absence of local anaesthesia as the current injection material (+ 0.34). Baseline pain VAS (+ 0.24) and younger age (+ 0.20) contributed additional positive increments, while baseline OHIP-14 score (scaled value = 0.916) and Saline non-assignment each exerted minor negative pulls (− 0.12 each). All contributions accumulated from the population base value E[f(X)] = 0.051.

Patient B (predicted probability of success = 0.01; f(x) = − 4.602) presented the clinical mirror image. A near-normal baseline MMO (scaled value = 1.373) generated the largest single negative contribution (− 2.15 log-odds units), reflecting the ceiling effect whereby a patient already approaching normal range cannot plausibly register a ≥ 5 mm opening gain meeting the composite MCID. Botox assignment was the second largest negative driver (− 1.24), followed by below-average baseline pain VAS (− 0.42) and younger age (− 0.36) — both indicating a patient whose impairment at baseline was relatively mild. The absence of Magnesium Sulfate (− 0.31) and Saline (− 0.21) further reduced the predicted probability, while previous injection history (+ 0.05) and the absence of local anesthesia history (+ 0.07) contributed to small opposing terms. Critically, this patient’s predicted failure does not reflect treatment inadequacy — it reflects insufficient baseline severity to demonstrate improvement simultaneously across all three MCID thresholds, which is a genuine structural limitation of AND-logic composite endpoints applied to patients presenting with mild-to-moderate baseline impairment.

### Decision curve analysis

**Fig. 5:**
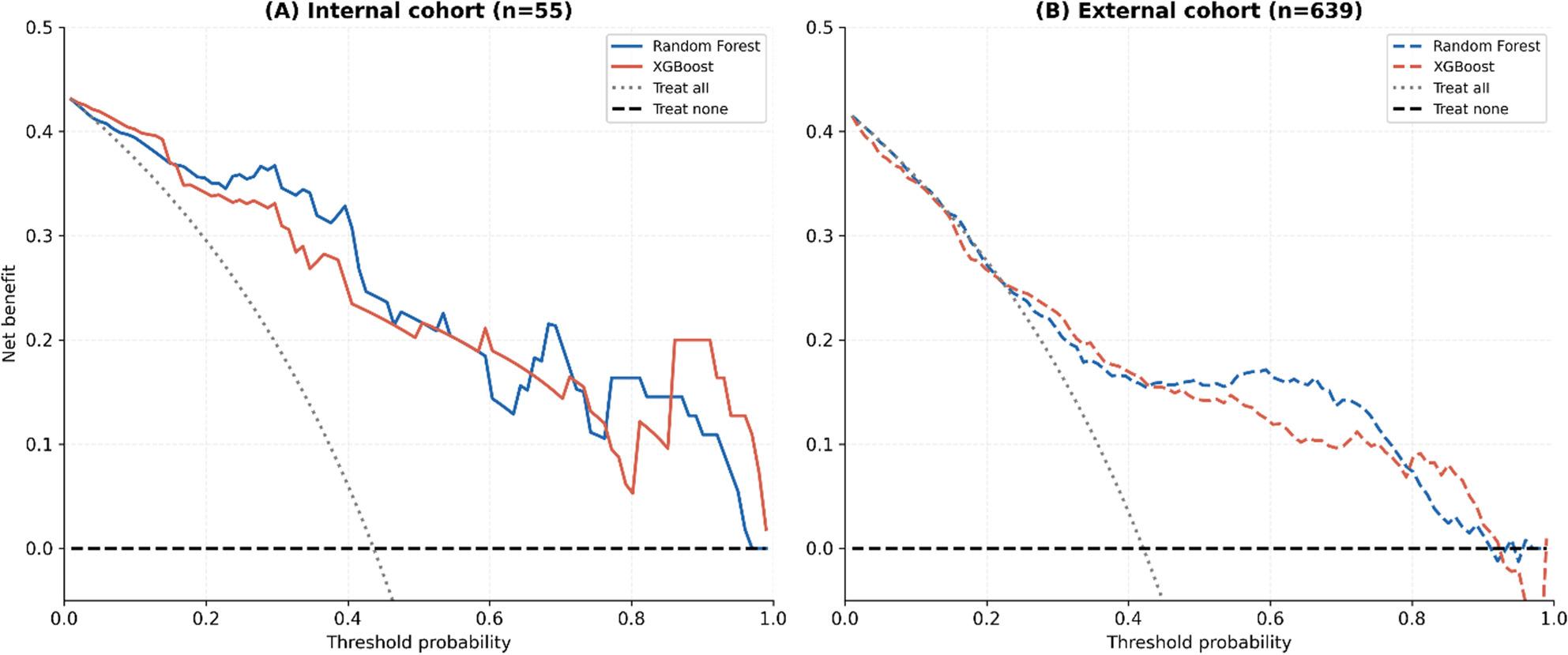
Decision curve analysis for random forest and XGBoost models across internal and external validation cohorts

To evaluate the clinical utility of both models beyond discrimination performance, Decision Curve Analysis (DCA) was performed across the full range of threshold probabilities for both the internal and external validation cohorts (Fig. 5). In the internal cohort (Panel A, *n* = 55), both Random Forest and XGBoost demonstrated positive net benefit across the entire clinically plausible threshold range, consistently outperforming both the “treat all” and “treat none” reference strategies. Random Forest showed a net benefit advantage over XGBoost particularly in the mid-to-high threshold range (approximately 0.30–0.85), suggesting superior clinical utility when clinicians apply more selective treatment criteria. In the external cohort (Panel B, *n* = 639), both models again maintained positive net benefit well above both reference strategies across all threshold probabilities, with Random Forest sustaining a clear advantage over XGBoost in the 0.40–0.90 threshold range. Both models crossed the zero net benefit line only at threshold probabilities above 0.95 — a range that carries no practical clinical relevance, as no clinician would require a 95% predicted probability of success before initiating TMJ injection therapy. These findings confirm that both models provide genuine clinical decision value across the full spectrum of risk tolerance, and that Random Forest offers the more conservative and consistently superior net benefit profile in high-threshold clinical scenarios.

## Discussion

The present study developed and externally validated two ensemble machine learning models, Random Forest and XGBoost, to predict multidimensional treatment success following trigger point injection therapy for masseter myofascial pain syndrome. Both models demonstrated excellent discrimination during internal validation and maintained acceptable performance in a geographically independent external cohort, supporting their potential generalizability across different clinical settings. Beyond predictive accuracy, the models consistently identified baseline maximum mouth opening, oral health-related quality of life, pain intensity, age, and injectable modality as the principal determinants of treatment outcome. The integration of SHAP-based explainability further enabled transparent interpretation of model predictions, while deployment of the validated XGBoost model as a web-based clinical decision support system represents an important step toward translating artificial intelligence into routine clinical decision-making for patients with myofascial pain.

Random Forest and XGBoost were selected because they are complementary ensemble learning algorithms that perform well on heterogeneous clinical datasets and provide transparent interpretation through SHAP analysis. The comparable performance of both models across the internal and external validation cohorts suggests that the identified predictor–outcome relationships represent robust clinical patterns rather than algorithm-specific behavior. This consistency strengthens confidence in the generalizability of the developed prediction framework and supports its potential application across different clinical settings. Furthermore, deployment of the validated XGBoost model as a web-based clinical decision support system enhances the translational value of the study by enabling individualized prediction of treatment success at the point of care. Together, these findings demonstrate how explainable machine learning can support more personalized, evidence-based treatment selection and represent an important step toward precision medicine in the management of orofacial myofascial pain [27–30].

Treatment success was defined using a composite endpoint requiring simultaneous clinically meaningful improvement in pain intensity, mandibular function, and oral health-related quality of life, with thresholds based on established MCIDs. This multidimensional outcome was selected because pain reduction alone does not necessarily reflect complete patient recovery; improvements in function and quality of life are equally important determinants of treatment success. Previous studies have similarly shown that reductions in pain do not consistently translate into sustained functional or quality-of-life gains, supporting the use of composite patient-centered outcomes in myofascial pain research [31–34].

The overall composite success rate of 43.1% should therefore be interpreted within the context of this stringent endpoint. Compared with studies evaluating single clinical outcomes, the present definition provides a more rigorous assessment of recovery and better reflects the heterogeneity of treatment response in masseter myofascial pain syndrome. From a predictive modelling perspective, this approach also creates a more clinically meaningful prediction task by identifying patients likely to achieve comprehensive recovery rather than isolated improvement in a single domain [35, 36].

From a predictive modeling perspective, the use of a stringent composite endpoint created a more clinically relevant prediction task by identifying factors associated with true multidimensional recovery rather than isolated improvements in a single outcome [37, 38].

A prominent finding of this study was the marked heterogeneity in treatment response across the six injectable modalities, indicating that treatment selection substantially influences clinical outcomes. Magnesium sulfate and platelet-rich fibrin (PRF) achieved the highest composite success rates, likely owing to their multimodal mechanisms of action. Magnesium sulfate modulates pain through N-methyl-D-aspartate (NMDA) receptor antagonism and attenuation of central sensitization, whereas PRF promotes tissue repair through the sustained release of growth factors [6, 39]. In contrast, botulinum toxin and local anesthetic injections demonstrated lower composite success. Although botulinum toxin effectively reduced pain, its limited improvement in mandibular function likely reduced the probability of achieving the composite endpoint, while local anesthetics appeared to provide mainly short-term symptom relief with less sustained functional and quality-of-life benefits [5, 9, 40]. These findings emphasize that both the biological mechanism of the injectable agent and the multidimensional nature of treatment success should be considered when selecting therapy for patients with masseter myofascial pain syndrome, highlighting the value of individualized treatment strategies [36].

To better understand treatment failure, a descriptive analysis of the non-responder subgroup was performed. Non-response varied substantially across injectable modalities, with the highest failure rates observed following local anesthetic and botulinum toxin injections, whereas magnesium sulfate and PRF showed markedly lower rates [5, 9, 39]. In contrast, baseline pain intensity and maximum mouth opening differed only minimally between responders and non-responders, suggesting that conventional measures of disease severity alone cannot adequately explain treatment variability [36]. These findings support the multifactorial nature of treatment response and underscore the need for multivariable machine learning models capable of integrating complex interactions among demographic, clinical, and treatment-related factors. The good discriminative performance achieved by both models despite the limited separation in baseline clinical characteristics further highlights the value of individualized prediction using multiple patient variables [41, 42].

Both Random Forest and XGBoost showed strong predictive performance, achieving excellent internal validation discrimination (AUC > 0.88) consistent with prior ensemble learning studies in complex clinical datasets [43–45]. The between-model difference was not significant, indicating comparable capability. As expected, predictive performance declined during external validation, reflecting the greater heterogeneity in patient populations, clinical practice, and data distributions across independent cohorts [46, 47]. Despite this reduction, both models retained acceptable-to-good discrimination, calibration, and precision-recall performance in the geographically independent cohort, confirming that the predictor-outcome relationships generalized beyond the development dataset. This modest performance decline supports model robustness and underscores why external validation remains the gold standard for assessing clinical applicability [48].

The attenuation in performance from training to external validation reflects some degree of model optimism, which is expected when applying machine learning to heterogeneous clinical data. Despite this, both Random Forest and XGBoost maintained acceptable-to-good discrimination, calibration, precision-recall performance, and positive net benefit on decision curve analysis in the geographically independent cohort, suggesting that the learned predictor-outcome relationships remained clinically meaningful despite some residual overfitting [45, 49].

The inclusion of a geographically independent external validation cohort—comprising patients from multiple institutions distinct from the development site—represents a major methodological strength. Unlike internal validation, which often overestimates real-world accuracy, external validation provides a more rigorous assessment of transportability and robustness [48, 50]. Notably, external validation remains uncommon in dental machine learning research, where most studies rely on single-center datasets and internal approaches such as cross-validation or random splitting limitation that has been identified as a major barrier to clinical implementation [43, 48]. In the present study, both models maintained acceptable predictive performance in the geographically distinct cohort, confirming that the identified predictor-outcome relationships were not site-specific artifacts. This finding enhances confidence in model generalizability and supports their potential utility as clinical decision-support tools across diverse practice environments. The consistent predictor rankings observed across validation cohorts further support the generalizability of our findings [51].

While ROC-AUC demonstrated good discriminative ability, additional evaluation using PR-AUC, calibration, and Decision Curve Analysis provided a more comprehensive assessment of model performance. Favorable results across these complementary metrics indicate that the models not only distinguished responders from non-responders but also generated well-calibrated predictions with potential clinical utility across relevant decision thresholds. Collectively, these findings strengthen confidence in the robustness and real-world applicability of the proposed prediction models beyond discrimination alone [46, 48, 50]. 

Despite the increasing predictive capabilities of machine learning algorithms, their adoption in clinical practice remains constrained by concerns regarding interpretability and transparency. Models such as Random Forest and XGBoost are often considered “black box” systems because their internal decision-making processes are not readily understandable to clinicians, potentially limiting trust, accountability, and acceptance in patient care [29]. To address this challenge, the present study employed SHapley Additive exPlanations (SHAP), an increasingly adopted interpretability framework grounded in cooperative game theory. SHAP quantifies both the magnitude and direction of each predictor’s contribution to an individual prediction while simultaneously providing population-level assessments of feature importance. Unlike traditional variable importance measures, SHAP enables transparent visualization of how specific patient characteristics influence predicted outcomes, thereby enhancing model interpretability and facilitating clinical understanding [45, 52]. By integrating SHAP analyses into both internal and external validation cohorts, the present study sought to balance predictive accuracy with interpretability, an essential requirement for trustworthy clinical decision-support systems.

SHAP analysis revealed a remarkably consistent predictor hierarchy across both Random Forest and XGBoost models and across both validation cohorts. Baseline maximum mouth opening (MMO) emerged as the most influential predictor of treatment success, followed by baseline OHIP-14 score, baseline pain intensity, and injectable material. These findings suggest that treatment response is determined by a combination of functional limitation, symptom burden, patient characteristics, and therapeutic intervention rather than any single clinical factor. The strong predictive importance of baseline MMO warrants particular attention. SHAP analysis demonstrated that patients with greater baseline mouth opening generally had a lower predicted probability of achieving treatment success, likely reflecting a ceiling-effect phenomenon [45, 52]. Patients who already exhibited near-normal mandibular function had limited opportunity to achieve the ≥ 5 mm improvement required by the composite endpoint, whereas those with more restricted baseline mouth opening had greater potential for clinically meaningful functional gains. Thus, the predictive effect of baseline MMO appears to reflect both the biological capacity for improvement and the structure of the outcome definition. The predictor hierarchy remained highly stable across algorithms and geographically distinct validation cohorts. Feature importance rankings demonstrated strong concordance between Random Forest and XGBoost, while cross-site analyses showed minimal variation in the relative importance of the identified predictors. This consistency strengthens confidence that baseline MMO, OHIP-14 score, age, pain intensity, and injectable material represent genuine determinants of treatment response rather than model-specific artifacts or site-dependent effects. The reproducibility of these findings across models and populations enhances the robustness, reliability, and potential clinical applicability of the developed prediction framework [44, 45].

The inclusion of injectable modality as a predictor was deliberate and reflects the intended clinical purpose of the model. Unlike conventional prognostic models, which estimate outcomes independently of treatment allocation, the present model was designed to support individualized treatment selection by estimating the probability of achieving composite treatment success under a specified therapeutic intervention. Consequently, the model addresses a clinically actionable question: “ What is the likelihood of treatment success for a patient with a given clinical profile if a particular injectable modality is selected? “. Within this framework, treatment assignment represents a known and modifiable decision variable available at the time of prediction rather than a post-treatment outcome. Excluding injectable modality would preclude the generation of treatment-specific risk estimates and substantially limit the model’s utility for personalized clinical decision-making. SHAP analysis demonstrated that treatment modality was only one component of the predictive framework, alongside baseline maximum mouth opening, OHIP-14 score, age, and pain intensity, indicating that outcome prediction was driven by the interaction between patient-specific characteristics and therapeutic choice. Accordingly, the model should be interpreted as a treatment-selection decision support tool that provides individualized estimates of treatment success conditional upon a chosen intervention, thereby facilitating evidence-based and patient-centered therapeutic planning.

Several limitations should be considered when interpreting the findings of the present study. First, although the dataset was derived from randomized controlled trials, the current investigation represents a retrospective secondary analysis and is therefore subject to the inherent limitations of observational predictive modeling. Second, treatment success was evaluated at a three-month follow-up, which captures short- to intermediate-term outcomes but does not provide information regarding the long-term durability of treatment effects or the need for subsequent interventions. Third, several potentially important predictors were unavailable across all source datasets and therefore could not be incorporated into the models. These included psychosocial variables such as anxiety, depression, pain catastrophizing, and somatization, as well as clinical factors including bruxism, pain duration, and laterality of involvement, all of which have been implicated in the pathogenesis and prognosis of myofascial pain disorders [35, 53]. Furthermore, outcome assessment was conducted at the patient level, precluding per-muscle analyses, and procedural variables such as injection dose, number of trigger points treated, injection technique, and operator experience were not consistently available for inclusion. Consequently, prospective studies incorporating a broader range of biopsychosocial and procedural variables are needed to determine whether predictive performance can be further improved. In addition, although treatment allocation within the original studies was randomized, the pooled dataset combined participants from six independent randomized controlled trials conducted across multiple centers. While randomization substantially reduces treatment allocation bias within individual trials, residual heterogeneity related to differences in study protocols, patient populations, recruitment practices, and center-specific factors may still have influenced model development and performance. Nevertheless, the strong external validation results suggest that the identified predictor-outcome relationships remained robust despite these between-trial differences. Future prospective multicenter validation studies are warranted to confirm the transportability, calibration, and clinical utility of the developed models in broader patient populations.

Future studies should focus on prospective multicenter validation and longer follow-up periods to assess the durability of treatment response and confirm model generalizability. Incorporating psychosocial and behavioral factors, such as anxiety, depression, and bruxism, may further improve predictive performance. In addition, dynamic prediction models integrated with electronic health records could enable real-time risk estimation and clinical decision support. Ultimately, comparative treatment recommendation systems capable of estimating individualized probabilities of success across multiple therapeutic options may further advance precision medicine in the management of myofascial pain.

## Conclusion

In conclusion, the present study demonstrates that ensemble machine learning models can accurately predict composite treatment success following masseter trigger point injection therapy. Baseline maximum mouth opening, OHIP-14 score, pain intensity, age, and injectable modality emerged as the most influential predictors of treatment response. The successful external validation of both models, together with SHAP-based interpretability analyses, supports their robustness, transparency, and potential clinical applicability. These findings highlight the promise of explainable machine learning as a tool for individualized treatment selection and represent an important step toward precision medicine in the management of myofascial pain disorders.

## Supplementary Information

Supplementary Material 1.

Supplementary Material 2.

## Data Availability

The datasets used and/or analyzed during the current study are available from the corresponding author on reasonable request. For privacy reasons, however, individual data allowing for the identification of participants cannot be made available.

## Abbreviations

MPS: Myofascial Pain Syndrome
MTrPs: Myofascial Trigger Points
TMD: Temporomandibular Disorders
TPI: Trigger Point Injection
DC/TMD: Diagnostic Criteria for Temporomandibular Disorders
PRF: Platelet-Rich Fibrin
ML: Machine Learning
VAS: Visual Analogue Scale
MMO: Maximum Mouth Opening
OHIP-14: Oral Health Impact Profile-14
IQR: Interquartile Range
QoL: Quality of Life
RF: Random Forest
ROC-AUC: Receiver Operating Characteristic – Area Under the Curve
XGBoost: Extreme Gradient Boosting
CDSS: web-based Clinical Decision Support System
SHAP: SHapley Additive exPlanations
PR-AUC: Precision–Recall Area Under the Curve
DCA: Decision Curve Analysis
CDSS: Clinical Decision Support System
CI: Confidence Interval
NMDA: N-Methyl-D-Aspartate
RCT: Randomized Controlled Trial
TRIPOD + AI: Transparent Reporting of a Multivariable Prediction Model for Individual Prognosis or Diagnosis–Artificial Intelligence Extension
