# Supplementary material for: A machine learning approach to predict treatment response in myofascial pain patients receiving masseter trigger point injections
Source: BMC Oral Health. 2026 Aug 10;26:1399. doi: 10.1186/s12903-026-09491-0 (PMC13455472; doi:10.1186/s12903-026-09491-0)
Supplement: Supplementary file 1 [file 12903_2026_9491_MOESM1_ESM.docx]

Statistical Analysis — Myofascial Pain TPI Study

Table 1, Table 2, Normality Assessment, Effect Sizes

Reham Ragab

May 11, 2026

Table of Contents

[1. Overview of the Analytical Dataset	2](#_Toc229380279)

[2. Normality Assessment	2](#_Toc229380280)

[2.1 Shapiro-Wilk Test + Skewness and Kurtosis	2](#_Toc229380281)

[2.2 Q-Q Plots — Baseline Variables	3](#_Toc229380282)

[2.3 Q-Q Plots — 3-Month Outcomes	4](#_Toc229380283)

[3. Table 1 — Baseline Characteristics by Treatment Group	5](#_Toc229380284)

[4. Table 2 — Treatment Outcomes at 1-Month and 3-Month Follow-Up	7](#_Toc229380285)

[5. Treatment Success Rates by Group	9](#_Toc229380286)

[6. Baseline Variable Distributions by Treatment Group	10](#_Toc229380287)

[7. Post-Treatment Outcome Distributions	11](#_Toc229380288)

[8. Kruskal-Wallis Pairwise Comparisons (Post-hoc)	11](#_Toc229380289)

[9. Data Quality Summary	15](#_Toc229380290)

# 1. Overview of the Analytical Dataset

| **Item** | **Value** |
| --- | --- |
| Total patients | 1181 |
| Treatment success | 509 |
| Treatment failure | 672 |
| Success rate (%) | 43.1% |
| Missing Pain M1 | 1 patient |

**Data quality notes:**

- Three patients (IDs 56, 367, 545) had MMO M1/M3 recorded as 334.5 mm — corrected to 33.45 mm (decimal point error).
- One patient had missing Pain M1 (retained in analysis; M1 not used in outcome definition).
- Location standardised: Alamein and Sohag combined as **Alamein/Sohag** (same research centre group).

# 2. Normality Assessment

## 2.1 Shapiro-Wilk Test + Skewness and Kurtosis

| **Variable** | **N** | **Mean** | **SD** | **Median** | **IQR [Q1–Q3]** | **Skewness** | **Kurtosis** | **Shapiro-Wilk p** | **Normal?** |
| --- | --- | --- | --- | --- | --- | --- | --- | --- | --- |
| Age (years) | 1,181 | 34.97 | 11.66 | 34.0 | [25–41] | 0.997 | 3.437 | <0.001 | No |
| Pain VAS — Baseline (M0) | 1,181 | 7.11 | 1.04 | 7.0 | [6–8] | -0.339 | 2.576 | <0.001 | No |
| MMO — Baseline mm (M0) | 1,181 | 30.88 | 2.01 | 30.7 | [30.1–32] | -6.406 | 87.391 | <0.001 | No |
| OHIP-14 — Baseline (M0) | 1,181 | 38.89 | 3.42 | 39.0 | [36–42] | -0.006 | 2.433 | <0.001 | No |
| Pain VAS — 3-month (M3) | 1,181 | 0.59 | 0.97 | 0.0 | [0–1] | 2.920 | 15.843 | <0.001 | No |
| MMO — 3-month mm (M3) | 1,181 | 35.36 | 2.10 | 35.6 | [33.4–37.1] | -0.061 | 1.835 | <0.001 | No |
| OHIP-14 — 3-month (M3) | 1,181 | 1.81 | 2.94 | 2.0 | [0–2] | 6.756 | 58.740 | <0.001 | No |

**Interpretation:** The Shapiro-Wilk test is sensitive to minor deviations from normality in large samples (n > 1,000). Visual inspection of Q-Q plots and skewness/kurtosis values (presented below) confirm the distributional characteristics supporting use of non-parametric tests (Kruskal-Wallis).

## 2.2 Q-Q Plots — Baseline Variables

Q-Q plots for baseline continuous variables. Deviation from the diagonal line indicates departure from normality.

## 2.3 Q-Q Plots — 3-Month Outcomes

Q-Q plots for 3-month outcome variables.

# 3. Table 1 — Baseline Characteristics by Treatment Group

| **Characteristic** | **Overall** N = 1,181*^1^* | **Botox** N = 299*^1^* | **Dry Needling** N = 200*^1^* | **Local Anaesthesia** N = 250*^1^* | **Magnesium Sulfate** N = 201*^1^* | **PRF** N = 151*^1^* | **Saline** N = 80*^1^* | **p-value***^2^* |
| --- | --- | --- | --- | --- | --- | --- | --- | --- |
| **Age, years** | 34.0 [25.0–41.0] | 33.0 [25.0–36.0] | 34.0 [25.0–39.5] | 32.0 [25.0–39.0] | 35.0 [27.0–47.0] | 35.0 [28.0–47.0] | 33.0 [25.0–42.0] | **<0.001** |
| **Sex** |  |  |  |  |  |  |  | **<0.001** |
| Female | 1,092 (92%) | 288 (96%) | 189 (95%) | 223 (89%) | 173 (86%) | 141 (93%) | 78 (98%) |  |
| Male | 89 (7.5%) | 11 (3.7%) | 11 (5.5%) | 27 (11%) | 28 (14%) | 10 (6.6%) | 2 (2.5%) |  |
| **Centre / Location** |  |  |  |  |  |  |  | **<0.001** |
| Alamein/Sohag | 320 (27%) | 40 (13%) | 91 (46%) | 106 (42%) | 33 (16%) | 31 (21%) | 19 (24%) |  |
| Fayoum | 542 (46%) | 108 (36%) | 108 (54%) | 124 (50%) | 127 (63%) | 14 (9.3%) | 61 (76%) |  |
| Private Practice | 319 (27%) | 151 (51%) | 1 (0.5%) | 20 (8.0%) | 41 (20%) | 106 (70%) | 0 (0%) |  |
| **Previous injection** | 660 (56%) | 282 (94%) | 18 (9.0%) | 22 (8.8%) | 193 (96%) | 139 (92%) | 6 (7.5%) | **<0.001** |
| **Baseline Pain VAS (0–10)** |  |  |  |  |  |  |  | **<0.001** |
| 4 | 3 (0.3%) | 1 (0.3%) | 1 (0.5%) | 0 (0%) | 1 (0.5%) | 0 (0%) | 0 (0%) |  |
| 5 | 89 (7.5%) | 17 (5.7%) | 13 (6.5%) | 13 (5.2%) | 29 (14%) | 17 (11%) | 0 (0%) |  |
| 6 | 223 (19%) | 60 (20%) | 35 (18%) | 47 (19%) | 33 (16%) | 27 (18%) | 21 (26%) |  |
| 7 | 400 (34%) | 108 (36%) | 89 (45%) | 104 (42%) | 34 (17%) | 33 (22%) | 32 (40%) |  |
| 8 | 391 (33%) | 95 (32%) | 54 (27%) | 72 (29%) | 89 (44%) | 58 (38%) | 23 (29%) |  |
| 9 | 75 (6.4%) | 18 (6.0%) | 8 (4.0%) | 14 (5.6%) | 15 (7.5%) | 16 (11%) | 4 (5.0%) |  |
| **Baseline MMO, mm** | 30.7 [30.1–32.0] | 30.9 [30.4–32.0] | 30.7 [30.0–31.5] | 30.9 [30.3–32.0] | 30.5 [29.7–31.0] | 30.9 [29.7–32.8] | 31.1 [30.5–32.5] | **<0.001** |
| **Baseline OHIP-14 (0–56)** | 39.0 [36.0–42.0] | 37.0 [35.0–39.0] | 40.0 [37.0–44.0] | 37.0 [35.0–41.0] | 42.0 [39.0–42.0] | 40.0 [38.0–40.0] | 37.0 [36.0–42.0] | **<0.001** |
| *^1^*Median [Q1–Q3]; n (%) | | | | | | | | |
| *^2^*Kruskal-Wallis test for continuous variables; Chi-square test for categorical variables | | | | | | | | |

```
##
## ── Effect Sizes: Kruskal-Wallis (Eta-squared) ──
## a flextable object.
## col_keys: `Variable`, `eta_sq`, `magnitude`, `n`
## header has 1 row(s)
## body has 4 row(s)
## original dataset sample:
## 'data.frame':    4 obs. of  4 variables:
##  $ Variable : chr  "Age" "Baseline Pain VAS" "Baseline MMO" "Baseline OHIP-14"
##  $ eta_sq   : num  0.019 0 0.028 0.136
##  $ magnitude: Ord.factor w/ 3 levels "small"<"moderate"<..: 1 1 1 2
##  $ n        : int  1181 1181 1181 1181
##
## ── Effect Sizes: Chi-square (Cramér's V) ──
## a flextable object.
## col_keys: `Variable`, `CramersV`, `p_value`
## header has 1 row(s)
## body has 3 row(s)
## original dataset sample:
## 'data.frame':    3 obs. of  3 variables:
##  $ Variable: chr  "Sex" "Centre" "Previous injection"
##  $ CramersV: num  0.149 0.43 0.858
##  $ p_value : chr  "<0.001" "<0.001" "<0.001"
```

**Effect size interpretation (η²):** Small = 0.01–0.06; Medium = 0.06–0.14; Large > 0.14. **Cramér’s V interpretation:** Small = 0.10–0.30; Medium = 0.30–0.50; Large > 0.50.

# 4. Table 2 — Treatment Outcomes at 1-Month and 3-Month Follow-Up

| **Characteristic** | **Overall** N = 1,181*^1^* | **Botox** N = 299*^1^* | **Dry Needling** N = 200*^1^* | **Local Anaesthesia** N = 250*^1^* | **Magnesium Sulfate** N = 201*^1^* | **PRF** N = 151*^1^* | **Saline** N = 80*^1^* | **p-value***^2^* |
| --- | --- | --- | --- | --- | --- | --- | --- | --- |
| **Required 2nd injection** | 63 (5.3%) | 1 (0.3%) | 6 (3.0%) | 38 (15%) | 0 (0%) | 0 (0%) | 18 (23%) | **<0.001** |
| **Treatment outcome** |  |  |  |  |  |  |  | **<0.001** |
| Failure | 672 (57%) | 229 (77%) | 127 (64%) | 198 (79%) | 43 (21%) | 48 (32%) | 27 (34%) |  |
| Success | 509 (43%) | 70 (23%) | 73 (37%) | 52 (21%) | 158 (79%) | 103 (68%) | 53 (66%) |  |
| **Pain VAS at 1-month** |  |  |  |  |  |  |  | **<0.001** |
| 0 | 520 (44%) | 197 (66%) | 10 (5.0%) | 110 (44%) | 161 (80%) | 39 (26%) | 3 (3.8%) |  |
| 1 | 602 (51%) | 102 (34%) | 163 (82%) | 124 (50%) | 40 (20%) | 111 (74%) | 62 (78%) |  |
| 2 | 55 (4.7%) | 0 (0%) | 24 (12%) | 16 (6.4%) | 0 (0%) | 0 (0%) | 15 (19%) |  |
| 3 | 3 (0.3%) | 0 (0%) | 3 (1.5%) | 0 (0%) | 0 (0%) | 0 (0%) | 0 (0%) |  |
| **Pain VAS at 3-month** |  |  |  |  |  |  |  | **<0.001** |
| 0 | 712 (60%) | 283 (95%) | 30 (15%) | 69 (28%) | 186 (93%) | 134 (89%) | 10 (13%) |  |
| 1 | 343 (29%) | 16 (5.4%) | 161 (81%) | 76 (30%) | 15 (7.5%) | 17 (11%) | 58 (73%) |  |
| 2 | 69 (5.8%) | 0 (0%) | 8 (4.0%) | 57 (23%) | 0 (0%) | 0 (0%) | 4 (5.0%) |  |
| 3 | 45 (3.8%) | 0 (0%) | 0 (0%) | 45 (18%) | 0 (0%) | 0 (0%) | 0 (0%) |  |
| 5 | 1 (<0.1%) | 0 (0%) | 0 (0%) | 0 (0%) | 0 (0%) | 0 (0%) | 1 (1.3%) |  |
| 6 | 5 (0.4%) | 0 (0%) | 0 (0%) | 1 (0.4%) | 0 (0%) | 0 (0%) | 4 (5.0%) |  |
| 7 | 6 (0.5%) | 0 (0%) | 1 (0.5%) | 2 (0.8%) | 0 (0%) | 0 (0%) | 3 (3.8%) |  |
| **Pain reduction (M0–M3)** | 7.0 [6.0–8.0] | 7.0 [6.0–8.0] | 6.0 [6.0–7.0] | 6.0 [5.0–7.0] | 7.0 [6.0–8.0] | 7.0 [6.0–8.0] | 6.0 [5.0–7.0] | **<0.001** |
| **MMO at 1-month, mm** | 36.8 [34.1–38.2] | 34.5 [33.7–36.6] | 36.9 [34.0–38.3] | 34.2 [33.6–35.6] | 38.1 [37.1–39.1] | 38.1 [36.9–38.6] | 37.9 [37.6–39.4] | **<0.001** |
| **MMO at 3-month, mm** | 35.6 [33.4–37.1] | 33.9 [32.8–35.6] | 34.6 [33.3–36.3] | 33.7 [32.7–35.4] | 37.1 [35.9–37.8] | 36.9 [36.1–38.1] | 36.9 [36.3–38.2] | **<0.001** |
| **MMO increase (M3–M0), mm** | 4.5 [2.4–6.3] | 2.9 [1.7–4.9] | 4.1 [2.2–5.5] | 2.8 [1.6–4.5] | 6.4 [5.2–7.7] | 5.9 [4.6–7.6] | 5.7 [4.5–7.1] | **<0.001** |
| **OHIP-14 at 1-month** |  |  |  |  |  |  |  | 1 |
| 0 | 169 (14%) | 42 (14%) | 29 (15%) | 36 (14%) | 29 (14%) | 22 (15%) | 11 (14%) |  |
| 1 | 169 (14%) | 43 (14%) | 28 (14%) | 36 (14%) | 28 (14%) | 22 (15%) | 12 (15%) |  |
| 2 | 168 (14%) | 43 (14%) | 29 (15%) | 35 (14%) | 28 (14%) | 21 (14%) | 12 (15%) |  |
| 3 | 170 (14%) | 44 (15%) | 27 (14%) | 37 (15%) | 30 (15%) | 22 (15%) | 10 (13%) |  |
| 4 | 168 (14%) | 42 (14%) | 29 (15%) | 35 (14%) | 29 (14%) | 21 (14%) | 12 (15%) |  |
| 5 | 168 (14%) | 41 (14%) | 30 (15%) | 35 (14%) | 29 (14%) | 21 (14%) | 12 (15%) |  |
| 6 | 169 (14%) | 44 (15%) | 28 (14%) | 36 (14%) | 28 (14%) | 22 (15%) | 11 (14%) |  |
| **OHIP-14 at 3-month** | 2.0 [0.0–2.0] | 2.0 [1.0–3.0] | 1.5 [0.0–2.0] | 2.0 [0.0–3.0] | 1.0 [0.0–2.0] | 2.0 [0.0–2.0] | 2.5 [1.0–3.0] | **<0.001** |
| **OHIP-14 reduction (M0–M3)** | 37.0 [35.0–40.0] | 36.0 [34.0–38.0] | 40.0 [36.0–42.0] | 35.0 [32.0–39.0] | 39.0 [37.0–41.0] | 38.0 [36.0–40.0] | 35.0 [33.5–39.0] | **<0.001** |
| *^1^*n (%); Median [Q1–Q3] | | | | | | | | |
| *^2^*Kruskal-Wallis test for continuous; Chi-square test for categorical. Values: Median [IQR] or n (%). | | | | | | | | |

Effect Sizes for Outcome Comparisons (Kruskal-Wallis, η²)

| **Outcome Variable** | **Eta-squared (η²)** | **Magnitude** |
| --- | --- | --- |
| Pain VAS 3-month | 0.535 | large |
| MMO 3-month | 0.364 | large |
| OHIP-14 3-month | 0.018 | small |
| Pain reduction | 0.181 | large |
| MMO increase | 0.290 | large |
| OHIP-14 reduction | 0.153 | large |

# 5. Treatment Success Rates by Group

Treatment Success Rates by Group. Chi-square p<0.001; Cramér's V = 0.47

| **Injectable Agent** | **Total (n)** | **Success (n)** | **Failure (n)** | **Success Rate (%)** |
| --- | --- | --- | --- | --- |
| Botox | 299 | 70 | 229 | 23.4 |
| Dry Needling | 200 | 73 | 127 | 36.5 |
| Local Anaesthesia | 250 | 52 | 198 | 20.8 |
| Magnesium Sulfate | 201 | 158 | 43 | 78.6 |
| PRF | 151 | 103 | 48 | 68.2 |
| Saline | 80 | 53 | 27 | 66.2 |

Treatment success rates by injectable agent with 95% confidence intervals (Wilson method).

# 6. Baseline Variable Distributions by Treatment Group

Boxplots of baseline clinical variables by treatment group.

# 7. Post-Treatment Outcome Distributions

Boxplots of change scores (delta) from baseline to 3 months by treatment group.

# 8. Kruskal-Wallis Pairwise Comparisons (Post-hoc)

```
##
## Baseline Pain VAS: No significant pairwise differences after Bonferroni correction.
##
## Baseline MMO — Significant pairwise differences (Dunn test, Bonferroni-adjusted):
## a flextable object.
## col_keys: `group1`, `group2`, `statistic`, `p`, `p.adj`, `p.adj.signif`
## header has 1 row(s)
## body has 5 row(s)
## original dataset sample:
## 'data.frame':    5 obs. of  6 variables:
##  $ group1      : chr  "Botox" "Dry Needling" "Local Anaesthesia" "Magnesium Sulfate" ...
##  $ group2      : chr  "Magnesium Sulfate" "Saline" "Magnesium Sulfate" "Saline" ...
##  $ statistic   : num  -4.48 3.56 -4.04 5.48 3.02
##  $ p           : num  7.53e-06 3.72e-04 5.30e-05 4.28e-08 2.52e-03
##  $ p.adj       : num  1.13e-04 5.59e-03 7.95e-04 6.42e-07 3.79e-02
##  $ p.adj.signif: chr  "***" "**" "***" "****" ...
##  - attr(*, "na.action")= 'omit' Named int [1:10] 6 11 12 16 17 18 21 22 23 24
##   ..- attr(*, "names")= chr [1:10] "6" "11" "12" "16" ...
##  - attr(*, "args")=List of 5
##   ..$ data           :'data.frame':  1181 obs. of  27 variables:
##   .. ..$ patient_id    : int [1:1181] 1 2 3 4 5 6 7 8 9 10 ...
##   .. ..$ age           : int [1:1181] 20 23 52 54 65 41 32 35 20 21 ...
##   .. ..$ sex           : chr [1:1181] "Female" "Female" "Male" "Female" ...
##   .. ..$ med_history   : chr [1:1181] "0" "0" "0" "Diabetic" ...
##   .. ..$ location      : chr [1:1181] "Fayoum" "Fayoum" "Fayoum" "Fayoum" ...
##   .. ..$ material      : Factor w/ 6 levels "Botox","Dry Needling",..: 3 3 3 3 3 3 3 3 3 3 ...
##   .. ..$ prev_injection: chr [1:1181] "No" "No" "No" "No" ...
##   .. ..$ prev_material : chr [1:1181] "0" "0" "0" "0" ...
##   .. ..$ need_2nd      : chr [1:1181] "No" "No" "No" "No" ...
##   .. ..$ time_2nd      : int [1:1181] 0 0 0 0 3 0 0 0 0 3 ...
##   .. ..$ muscle        : chr [1:1181] "Masseter" "Masseter" "Masseter" "Masseter" ...
##   .. ..$ adjunctive    : chr [1:1181] "Appliance " "Appliance " "Appliance " "Appliance " ...
##   .. ..$ complications : chr [1:1181] "no" "no" "no" "no" ...
##   .. ..$ pain_m0       : int [1:1181] 7 6 6 8 9 7 8 6 5 8 ...
##   .. ..$ pain_m1       : int [1:1181] 2 1 2 2 2 1 0 2 1 0 ...
##   .. ..$ pain_m3       : int [1:1181] 2 1 2 2 1 1 0 2 1 0 ...
##   .. ..$ mmo_m0        : num [1:1181] 30 31 32 33 33.5 25.5 29.5 28.5 27 28 ...
##   .. ..$ mmo_m1        : num [1:1181] 35.5 35.6 35.4 35.6 34.9 34.7 36 35.5 35 34 ...
##   .. ..$ mmo_m3        : num [1:1181] 35.5 35.5 35.4 35.6 34.9 34.7 35.4 35.5 35 30.9 ...
##   .. ..$ ohip_m0       : int [1:1181] 40 41 42 43 39 32 34 33 41 49 ...
##   .. ..$ ohip_m1       : int [1:1181] 3 0 4 5 2 1 6 3 0 4 ...
##   .. ..$ ohip_m3       : int [1:1181] 2 0 2 0 2 0 2 0 2 16 ...
##   .. ..$ delta_pain    : int [1:1181] 5 5 4 6 8 6 8 4 4 8 ...
##   .. ..$ delta_mmo     : num [1:1181] 5.5 4.5 3.4 2.6 1.4 ...
##   .. ..$ delta_ohip    : int [1:1181] 38 41 40 43 37 32 32 33 39 33 ...
##   .. ..$ success       : int [1:1181] 1 0 0 0 0 1 1 1 1 0 ...
##   .. ..$ success_label : chr [1:1181] "Success" "Failure" "Failure" "Failure" ...
##   ..$ formula        :Class 'formula'  language mmo_m0 ~ material
##   .. .. ..- attr(*, ".Environment")=<environment: 0x000002234091a650>
##   ..$ p.adjust.method: chr "bonferroni"
##   ..$ detailed       : logi FALSE
##   ..$ method         : chr "dunn_test"
##
## Baseline OHIP-14 — Significant pairwise differences (Dunn test, Bonferroni-adjusted):
## a flextable object.
## col_keys: `group1`, `group2`, `statistic`, `p`, `p.adj`, `p.adj.signif`
## header has 1 row(s)
## body has 9 row(s)
## original dataset sample:
## 'data.frame':    9 obs. of  6 variables:
##  $ group1      : chr  "Botox" "Botox" "Botox" "Dry Needling" ...
##  $ group2      : chr  "Dry Needling" "Magnesium Sulfate" "PRF" "Local Anaesthesia" ...
##  $ statistic   : num  8.44 8.36 4.5 -9.44 -2.99 ...
##  $ p           : num  3.05e-17 6.02e-17 6.95e-06 3.70e-21 2.76e-03 ...
##  $ p.adj       : num  4.57e-16 9.03e-16 1.04e-04 5.55e-20 4.15e-02 ...
##  $ p.adj.signif: chr  "****" "****" "***" "****" ...
##  - attr(*, "na.action")= 'omit' Named int [1:10] 6 11 12 16 17 18 21 22 23 24
##   ..- attr(*, "names")= chr [1:10] "6" "11" "12" "16" ...
##  - attr(*, "args")=List of 5
##   ..$ data           :'data.frame':  1181 obs. of  27 variables:
##   .. ..$ patient_id    : int [1:1181] 1 2 3 4 5 6 7 8 9 10 ...
##   .. ..$ age           : int [1:1181] 20 23 52 54 65 41 32 35 20 21 ...
##   .. ..$ sex           : chr [1:1181] "Female" "Female" "Male" "Female" ...
##   .. ..$ med_history   : chr [1:1181] "0" "0" "0" "Diabetic" ...
##   .. ..$ location      : chr [1:1181] "Fayoum" "Fayoum" "Fayoum" "Fayoum" ...
##   .. ..$ material      : Factor w/ 6 levels "Botox","Dry Needling",..: 3 3 3 3 3 3 3 3 3 3 ...
##   .. ..$ prev_injection: chr [1:1181] "No" "No" "No" "No" ...
##   .. ..$ prev_material : chr [1:1181] "0" "0" "0" "0" ...
##   .. ..$ need_2nd      : chr [1:1181] "No" "No" "No" "No" ...
##   .. ..$ time_2nd      : int [1:1181] 0 0 0 0 3 0 0 0 0 3 ...
##   .. ..$ muscle        : chr [1:1181] "Masseter" "Masseter" "Masseter" "Masseter" ...
##   .. ..$ adjunctive    : chr [1:1181] "Appliance " "Appliance " "Appliance " "Appliance " ...
##   .. ..$ complications : chr [1:1181] "no" "no" "no" "no" ...
##   .. ..$ pain_m0       : int [1:1181] 7 6 6 8 9 7 8 6 5 8 ...
##   .. ..$ pain_m1       : int [1:1181] 2 1 2 2 2 1 0 2 1 0 ...
##   .. ..$ pain_m3       : int [1:1181] 2 1 2 2 1 1 0 2 1 0 ...
##   .. ..$ mmo_m0        : num [1:1181] 30 31 32 33 33.5 25.5 29.5 28.5 27 28 ...
##   .. ..$ mmo_m1        : num [1:1181] 35.5 35.6 35.4 35.6 34.9 34.7 36 35.5 35 34 ...
##   .. ..$ mmo_m3        : num [1:1181] 35.5 35.5 35.4 35.6 34.9 34.7 35.4 35.5 35 30.9 ...
##   .. ..$ ohip_m0       : int [1:1181] 40 41 42 43 39 32 34 33 41 49 ...
##   .. ..$ ohip_m1       : int [1:1181] 3 0 4 5 2 1 6 3 0 4 ...
##   .. ..$ ohip_m3       : int [1:1181] 2 0 2 0 2 0 2 0 2 16 ...
##   .. ..$ delta_pain    : int [1:1181] 5 5 4 6 8 6 8 4 4 8 ...
##   .. ..$ delta_mmo     : num [1:1181] 5.5 4.5 3.4 2.6 1.4 ...
##   .. ..$ delta_ohip    : int [1:1181] 38 41 40 43 37 32 32 33 39 33 ...
##   .. ..$ success       : int [1:1181] 1 0 0 0 0 1 1 1 1 0 ...
##   .. ..$ success_label : chr [1:1181] "Success" "Failure" "Failure" "Failure" ...
##   ..$ formula        :Class 'formula'  language ohip_m0 ~ material
##   .. .. ..- attr(*, ".Environment")=<environment: 0x0000022352513698>
##   ..$ p.adjust.method: chr "bonferroni"
##   ..$ detailed       : logi FALSE
##   ..$ method         : chr "dunn_test"
##
## Age — Significant pairwise differences (Dunn test, Bonferroni-adjusted):
## a flextable object.
## col_keys: `group1`, `group2`, `statistic`, `p`, `p.adj`, `p.adj.signif`
## header has 1 row(s)
## body has 3 row(s)
## original dataset sample:
## 'data.frame':    3 obs. of  6 variables:
##  $ group1      : chr  "Botox" "Botox" "Local Anaesthesia"
##  $ group2      : chr  "Magnesium Sulfate" "PRF" "PRF"
##  $ statistic   : num  4.03 4.19 3.09
##  $ p           : num  5.64e-05 2.75e-05 2.03e-03
##  $ p.adj       : num  0.000846 0.000413 0.030402
##  $ p.adj.signif: chr  "***" "***" "*"
##  - attr(*, "na.action")= 'omit' Named int [1:10] 6 11 12 16 17 18 21 22 23 24
##   ..- attr(*, "names")= chr [1:10] "6" "11" "12" "16" ...
##  - attr(*, "args")=List of 5
##   ..$ data           :'data.frame':  1181 obs. of  27 variables:
##   .. ..$ patient_id    : int [1:1181] 1 2 3 4 5 6 7 8 9 10 ...
##   .. ..$ age           : int [1:1181] 20 23 52 54 65 41 32 35 20 21 ...
##   .. ..$ sex           : chr [1:1181] "Female" "Female" "Male" "Female" ...
##   .. ..$ med_history   : chr [1:1181] "0" "0" "0" "Diabetic" ...
##   .. ..$ location      : chr [1:1181] "Fayoum" "Fayoum" "Fayoum" "Fayoum" ...
##   .. ..$ material      : Factor w/ 6 levels "Botox","Dry Needling",..: 3 3 3 3 3 3 3 3 3 3 ...
##   .. ..$ prev_injection: chr [1:1181] "No" "No" "No" "No" ...
##   .. ..$ prev_material : chr [1:1181] "0" "0" "0" "0" ...
##   .. ..$ need_2nd      : chr [1:1181] "No" "No" "No" "No" ...
##   .. ..$ time_2nd      : int [1:1181] 0 0 0 0 3 0 0 0 0 3 ...
##   .. ..$ muscle        : chr [1:1181] "Masseter" "Masseter" "Masseter" "Masseter" ...
##   .. ..$ adjunctive    : chr [1:1181] "Appliance " "Appliance " "Appliance " "Appliance " ...
##   .. ..$ complications : chr [1:1181] "no" "no" "no" "no" ...
##   .. ..$ pain_m0       : int [1:1181] 7 6 6 8 9 7 8 6 5 8 ...
##   .. ..$ pain_m1       : int [1:1181] 2 1 2 2 2 1 0 2 1 0 ...
##   .. ..$ pain_m3       : int [1:1181] 2 1 2 2 1 1 0 2 1 0 ...
##   .. ..$ mmo_m0        : num [1:1181] 30 31 32 33 33.5 25.5 29.5 28.5 27 28 ...
##   .. ..$ mmo_m1        : num [1:1181] 35.5 35.6 35.4 35.6 34.9 34.7 36 35.5 35 34 ...
##   .. ..$ mmo_m3        : num [1:1181] 35.5 35.5 35.4 35.6 34.9 34.7 35.4 35.5 35 30.9 ...
##   .. ..$ ohip_m0       : int [1:1181] 40 41 42 43 39 32 34 33 41 49 ...
##   .. ..$ ohip_m1       : int [1:1181] 3 0 4 5 2 1 6 3 0 4 ...
##   .. ..$ ohip_m3       : int [1:1181] 2 0 2 0 2 0 2 0 2 16 ...
##   .. ..$ delta_pain    : int [1:1181] 5 5 4 6 8 6 8 4 4 8 ...
##   .. ..$ delta_mmo     : num [1:1181] 5.5 4.5 3.4 2.6 1.4 ...
##   .. ..$ delta_ohip    : int [1:1181] 38 41 40 43 37 32 32 33 39 33 ...
##   .. ..$ success       : int [1:1181] 1 0 0 0 0 1 1 1 1 0 ...
##   .. ..$ success_label : chr [1:1181] "Success" "Failure" "Failure" "Failure" ...
##   ..$ formula        :Class 'formula'  language age ~ material
##   .. .. ..- attr(*, ".Environment")=<environment: 0x00000223514ba470>
##   ..$ p.adjust.method: chr "bonferroni"
##   ..$ detailed       : logi FALSE
##   ..$ method         : chr "dunn_test"
## [[1]]
## NULL
##
## [[2]]
## NULL
##
## [[3]]
## NULL
##
## [[4]]
## NULL
```

# 9. Data Quality Summary

Data Quality Issues and Corrections

| **Variable** | **Issue** | **Action** |
| --- | --- | --- |
| MMO M1/M3 (patients 56, 367, 545) | Value 334.5 — likely decimal error | Corrected to 33.45 mm |
| Pain M1 (1 patient) | 1 missing value | Retained (not used in primary outcome) |
| Location standardisation | Fayoum/fayoum merged; Sohag merged with Alamein | Standardised to 3 centres |
| Implausible values clipped | MMO clipped 0–80 mm; Pain 0–10; OHIP-14 0–56 | Applied during preprocessing |

*Document generated automatically from AI_injection.csv using R 4.6.0.* *Packages: tidyverse, rstatix, ggpubr, gtsummary, flextable, moments.*
